# Establishment of a human 3D in vitro liver-bone model as a potential system for drug toxicity screening

**DOI:** 10.1007/s00204-024-03899-9

**Published:** 2024-11-06

**Authors:** Guanqiao Chen, Yuxuan Xin, Mohammad Majd Hammour, Bianca Braun, Sabrina Ehnert, Fabian Springer, Massoud Vosough, Maximilian M. Menger, Ashok Kumar, Andreas K. Nüssler, Romina H. Aspera-Werz

**Affiliations:** 1 Department of Traumatology, Siegfried Weller Institute, BG-Klinik Tübingen, Eberhard Karls University, 72076 Tübingen, Germany; 2 Department of Radiology, BG-Klinik Tübingen, Eberhard Karls University, 72076 Tübingen, Germany; 3 Department of Diagnostic and Interventional Radiology, University Hospital Tübingen, Hoppe-Seyler-Str. 3, 72076 Tübingen, Germany; 4 Department of Regenerative Medicine, Cell Science Research Center, Royan Institute for Stem Cell Biology and Technology, ACECR, Tehran, 1665659911 Iran; 5 Experimental Cancer Medicine, Institution for Laboratory Medicine, Karolinska Institutet, Stockholm, Sweden; 6 Biomaterial and Tissue Engineering Group, Department of Biological Sciences and Bioengineering, Indian Institute of Technology Kanpur, Kanpur, 208016 India; 7 Centre for Nanosciences, Indian Institute of Technology Kanpur, Kanpur, 208016 India; 8 Centre for Environmental Sciences and Engineering, Indian Institute of Technology Kanpur, Kanpur, 208016 India

**Keywords:** 3D in vitro liver-bone co-culture model, Diclofenac, Drug affect bone homeostasis, Liver-bone axis, Reactive Oxygen Species, Interleukin-6

## Abstract

Drug toxicity is an important cause of chronic liver damage, which in the long term can lead to impaired bone homeostasis through an imbalance in the liver-bone axis. For instance, non-steroidal anti-inflammatory drugs (*e.g.,* diclofenac), which are commonly used to control pain during orthopaedic interventions, are known to reduce bone quality and are the most prevalent causes of drug-induced liver damage. Therefore, we used human cell lines to produce a stable, reproducible, and reliable in vitro liver-bone co-culture model, which mimics the impaired bone homeostasis seen after diclofenac intake in vivo. To provide the best cell culture conditions for the two systems, we tested the effects of supplements contained in liver and bone cell culture medium on liver and bone cell lines, respectively. Additionally, different ratios of culture medium combinations on bone cell scaffolds and liver spheroids’ viability and function were also analysed. Then, liver spheroids and bone scaffolds were daily exposed to 3–6 µM diclofenac alone or in co-culture to compare and evaluate its effect on the liver and bone system. Our results demonstrated that a 50:50 liver:bone medium combination maintains the function of liver spheroids and bone scaffolds for up to 21 days. Osteoclast-like cell activity was significantly upregulated after chronic exposure to diclofenac only in bone scaffolds co-cultured with liver spheroids. Consequently, the mineral content and stiffness of bone scaffolds treated with diclofenac in co-culture with liver spheroids were significantly reduced. Interestingly, our results show that the increase in osteoclastic activity in the system is not related to the main product of diclofenac metabolism. However, osteoclast activation correlated with the increase in oxidative stress and inflammation associated with chronic diclofenac exposure. In summary, we established a long-term stable liver-bone system that represents the interaction between the two organs, meanwhile, it is also an outstanding model for studying the toxicity of drugs on bone homeostasis.

**Supplementary Information:**

The online version contains supplementary material available at 10.1007/s00204-024-03899-9.

## Introduction

Drug-induced osteoporosis is a prevalent condition that exerts a substantial impact on the quality of patients’ lives. For example, heparin, glucocorticoids, and thiazolidinediones, which are frequently used in clinical, have been confirmed can affect bone homeostasis and increase fracture risk (Mazziotti et al. 2010; Tannirandorn and Epstein 2000). After undergoing liver metabolism, the toxicity of drugs and their metabolites, along with disruptions in the liver-bone axis, could affect on bone homeostasis (Jin et al. 2022; Lu et al. 2022).

Recent studies have shown that the non-steroidal anti-inflammatory drug (NSAID), diclofenac, which is commonly used to control pain during orthopaedic interventions could cause liver damage and affect bone homeostasis (Cottrell and O’Connor 2010; Karanikola et al. 2022). When diclofenac enters the body, it undergoes metabolism in the liver by hepatocyte CYP enzymes to form 4′-hydroxy-, 3′-hydroxy-, and 5′-hydroxy-diclofenac, which then travel through the bloodstream to reach several organs of the body. In our previous study, we used aged mice as a model to confirm that diclofenac toxicity on bone homeostasis and has a negative effect on bone fracture healing (Menger et al. 2023).

While an expanding array of drugs has been identified for their potential to disrupt bone homeostasis with prolonged usage, the underlying cause and specific mechanisms remain unclear. Further research is needed to elucidate the intricate pathways and factors contributing to this phenomenon. Animal models have been used as sentinels for the investigative toxicology of drugs. Millions of animals are used in experimental research every year around the world (Doke and Dhawale 2015). However, animal experiments have certain limitations that need to be considered. Besides the high cost, time-consuming nature, and ethical concerns, they can also be difficult to reproduce, particularly when the experimental conditions are complex. Meanwhile, mechanical stress on bone tissue and drug metabolism are different in humans and animals, meaning that the results obtained from animal experiments may not be directly applicable to humans (McGonigle and Ruggeri 2014). Nevertheless, there is currently no in vitro model that represents the interaction between liver and bone tissue.

For many years, researchers have attempted to develop in vitro liver models based on 2D systems. However, 2D cell models do not accurately replicate the complex three-dimensional architecture and cellular interactions found in living tissues, which can limit their predictive power. Also, 2D cell models may not accurately predict drug efficacy or toxicity in vivo, which can limit their use in drug discovery and development. HepaRG cell line represents a successful model for hepatotoxicity studies and our preliminary data has also demonstrated that HepaRG cells showed a metabolic capacity comparable to that of human hepatocytes (Hammour et al. 2022). Therefore in this study, to improve the metabolic performance of HepaRG cells, spheroids 3D cultures were established to represent the liver. Regarding bone tissue, the cryogel technique was employed to manufacture a scaffold that contains human platelet-rich plasma (hPRP) as a component of the organic bone matrix. The appropriate pore size and stiffness make this scaffold an ideal platform for this application (Weng et al. 2020). As bone resorbing–forming progenitor cells, the human monocytic-like cells (THP-1) and bone-marrow-derived immortalised human mesenchymal stem cells (SCP-1) were used to seed the scaffold.

In this study, we demonstrated the manufacturing and optimisation process of a 3D in vitro liver-bone co-culture model. Furthermore, we present diclofenac to validate the in vitro system, and to investigate how diclofenac affects bone homeostasis. This new 3D co-culture model of bone and liver-like cells mimics in vivo conditions and provides a tool for screening the toxic effects of drugs on bone cells and for better study of the molecular pathogenesis and potential of the liver-bone axis interactions. Moreover, this model is closer to human liver-bone interaction and will contribute to reducing animal experiments.

## Materials and methods

### Cell lines

The human hepatocellular carcinoma cell line, HepaRG (Biopredic International, Saint Grégoire, France), was maintained in an undifferentiated state using HepaRG cells culture medium for 2 weeks (proliferation phase), which consisted of William’s Medium E (AL240, Omni Life Science) supplemented with 10% fetal calf serum (FCS; 10270-106, Life Technologies), 5 µg/mL insulin (Novo Nordisk), 2 mM glutamine (M11-006, Sigma), 50 µM hydrocortisone (Pfizer), and 1% Penicillin/Streptomycin (Pen/Strep; P0781, Sigma). The cells were incubated in a humidified cell incubator at 37 °C and 5% CO_2_, with medium changes performed three times a week. Differentiation of the cells was initiated by the addition of 1.7% dimethyl sulfoxide (DMSO; 4720.2, Roth).

SCP-1, a mesenchymal stem cell line (kindly provided by Prof. Dr. Matthias Schieker (Bocker et al. 2008)) was used as osteoprogenitor cells and cultured in Minimum Essential Medium Eagle Alpha (MEM α; AL081A, Omni Life Science) supplemented with 5% FCS at 37 °C in a humidified atmosphere with 5% CO_2_. The growing medium was refreshed twice weekly.

The human leukaemia monocytic cell line, THP-1 cells (ACC16, DSMZ) were used as the osteoclastic precursor cells, which were cultured as a suspension cell culture in RPMI 1640 Medium (R8758, Sigma) supplemented with 5% FCS. The cells were maintained in a humidified atmosphere with 5% CO_2_ at 37 °C, and the growing medium was renewed twice weekly.

### Human platelet-rich plasma (hPRP) scaffolds manufacturing and sterilisation

PRP scaffolds were generated as previously described (Haussling et al. 2019). To prepare the PRP, EDTA blood samples were collected from a group of five healthy volunteers and then centrifuged at 1000 × g for 10 min. A mixture was then prepared by combining an aqueous solution containing 16.0% pHEMA (128635-500G, Sigma), 0.3% Bis-Acrylamide (3039.1, Carl Roth), and 0.25 g/L PRP, this mixture was carefully stirred and cooled in ice. Following 30 min of incubation, di-sodium hydrogen phosphate buffer (T876.1, Carl Roth) was added to the solution to achieve a final concentration of 0.3 M. Immediately after this step, a mixture of 0.1% glutaraldehyde (3778.1, Carl Roth), 0.2% Ammonium Persulfate (A3678-25G, Sigma), and 0.2% TEMED (2367.3, Carl Roth) was added and thoroughly mixed. The resulting solution was then dispensed into polystyrene casting molds, with each mold receiving 2 mL of the solution, and then immediately frozen at a temperature of -18 °C for at least 12 h. To facilitate the slicing of the formed matrix with a razor blade, it was deep-frozen at a temperature of -80 °C for 1 h. The resulting scaffolds had uniform dimensions, measuring 3 mm in height and 6 mm in diameter. Afterward, the scaffolds were immediately transferred to a 1 M CaCl_2_ (CN93.2, Carl Roth) solution to promote the crystallisation of calcium phosphate, specifically hydroxyapatite. Following a 24-h incubation period, the CaCl_2_ solution was carefully removed, and the scaffolds were washed with phosphate-buffered saline (PBS; L182-50, Merck) for 15 min. During this process, ice crystals were formed to serve as placeholders for creating the desired pores. The size and shape of the pores were affected by the temperature and speed at which the freezing occurs (Adnan Memic and Joseph Steingold 2019).

To eliminate the unreacted compounds that may potentially be toxic and to also achieve sterilisation, the PBS was completely removed from the scaffolds. Subsequently, the scaffolds were immersed in 70% ethanol and shaken for at least 12 h. Following this, the sterilised scaffolds underwent four washing steps using sterile PBS for 30, 60, 90, and 120 min, respectively. Afterward, the scaffolds were incubated in THP-1 cell growing medium under humidified conditions with 5% CO_2_ at a temperature of 37 °C for 48 h. This pre-conditioning process served as a sterility control.

### Agarose plate and microwells manufacturing and sterilisation

**Fig. 1:**
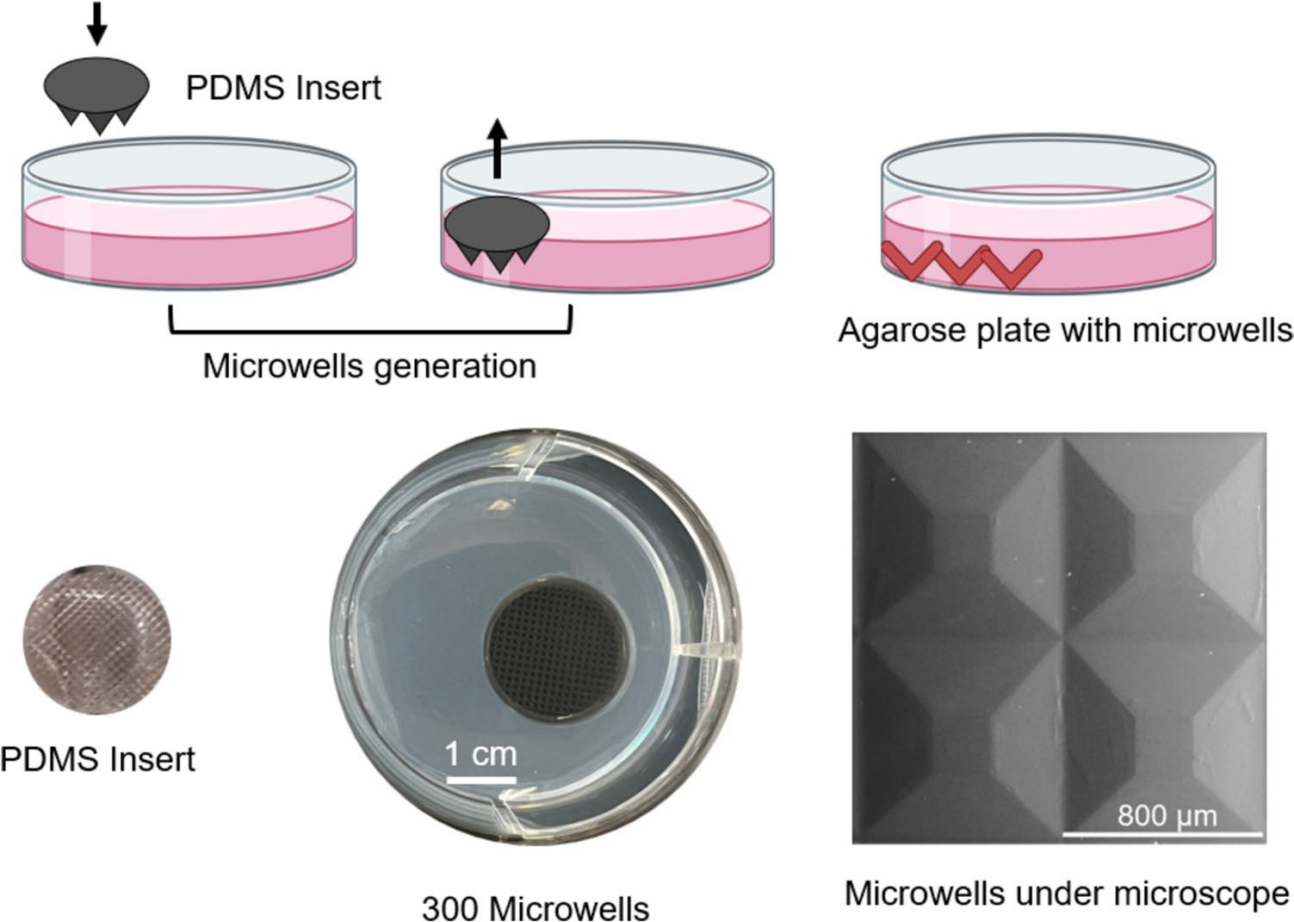
Agarose microwells generation process and their characteristics

To create non-adherent agarose microwell plates, mold-replication technology was used as previously described (Ghezelayagh et al. 2022). To produce the microwell plates, 3 mL melted 2% agarose (50004, Lonza) was poured in a well from a 6-well plate and with the help of a polydimethylsiloxane (PDMS) insert 300 pyramidal micro-wells, each with an 800 µm diameter were stamped. After the agarose solidified, the insert was gently removed, revealing a mirror-inverted patterned agarose surface within each well (Fig. 1). The plates were sterilized by an hour of exposure to ultraviolet light before use (Zahmatkesh et al. 2022).

### Cell seeding

#### Bone cells co-culture

For the 2D bone cells co-culture model, THP-1 cells (2.4 × 10^4^ cells per well) were seeded in the 96-well plate with 200 nM of phorbol 12-myristate 13-acetate (PMA; Cay10008014-1, Biomol) in 100 µL of THP-1 cells growing medium. After 24 h, the THP-1 cell growing medium was removed, and SCP-1 cells (0.3 × 10^4^ cells per well) were seeded into the same well with 100 µL bone differentiation medium (50:50 mix of RPMI and MEM α, 2% FCS, 200 µM L-ascorbic acid 2-phosphate (A8960-5G, Sigma), 5 mM β-glycerolphosphate (G9422-10, Sigma), 25 mM HEPES (HN78.2, Carl Roth), 1.5 mM CaCl_2_ and 5 µM cholecalciferol (95,230, Sigma). The bone co-culture system was cultured in the humidified conditions incubator at 37 °C with 5% CO_2_ and the bone differentiation medium was replaced twice a week.

For the 3D bone cell co-culture model, after sterile control, the THP-1 growing medium was totally aspirated from the scaffolds. A single scaffold was placed at the centre of each well in a 48-well plate. Following a previously described method (Weng et al. 2020), THP-1 cells (8 × 10^4^ cells/15 µL per scaffold) were seeded with 200 nM of PMA into the scaffold. Following 4 h of incubation at 37 °C, 5% CO_2_ humidified atmosphere, 500 µL THP-1 growing medium with 200 nM PMA was carefully added to each scaffold. The specimens were incubated in a humidified atmosphere with 5% CO_2_ at 37 °C for 24 h to ensure complete adherence. The following day, the THP-1 cell growing medium was removed before seeding. SCP-1 cells (1 × 10^4^ cells/15 µL per scaffold) were seeded into the same scaffold. After 4 h, 500 μL of bone differentiation medium was gently added (Haussling et al. 2021). The bone co-culture scaffolds were cultured in the humidified conditions incubator at 37 °C with 5% CO_2_ and the bone differentiation medium was replaced twice a week.

#### HepaRG cell culture

For the 2D liver model, HepaRG cells (0.9 × 10^4^ cells per well) with 100 µL HepaRG cell culture medium were seeded into the 96-well plate. After 2 weeks of culture, cell differentiation was induced with 1.7% of DMSO for 2 weeks before the cells were used in the experiment. Cells were cultured in a humidified incubator at 37 °C with 5% CO_2_ and the medium was changed three times per week.

For the liver spheroids model, before seeding the HepaRG cells, we added 2 mL HepaRG cell culture medium into each agarose well to create suitable conditions for cells and centrifugation. Then, HepaRG cells (30 × 10^4^ cells/mL per well, 1000 cells/microwell) were seeded into the wells to generate spheroids. Afterward, the plate was centrifuged (3 min, 1200 rpm) to make the cells distribute equally in the microwells. HepaRG cell distribution in the microwells was checked by light microscopy. The following day, cell differentiation was induced with 1.7% DMSO, and cells were cultured in the humidified incubator at 37 °C with 5% CO_2,_ and the HepaRG cell culture medium was changed three times per week.

### Investigating the influence of supplements contained in the bone and liver medium on the 2D bone and liver cells respectively

To evaluate the effect of supplements, contained in bone differentiation medium, on HepaRG cells, we treated HepaRG cells in 96-well culture plates with 25 mM HEPES, 1.5 mM Calcium chloride, 20 ng/mL Cholecalciferol, 200 µM L-Ascorbic acid 2-phosphate, and 5 mM β-Glycerolphosphate dissolved in HepaRG cell differentiation medium. Stimulation and medium change were done three times per week. Cell viability and function were measured on days 7, 14 and 21.

To evaluate the effect of supplements, contained in the HepaRG cell differentiation medium, on SCP-1 and THP-1 bone co-culture system, we seeded THP-1 and SCP-1 cells in 96-well culture plates (according to Sect. Bone cells co-culture). The following day the co-culture system was stimulated with 50 µM Hydrocortisone, 2 mM L-Glutamine, 1,7% DMSO, and 5 µg/ml Insulin dissolved in the bone differentiation medium. Stimulation and medium change were done twice a week. Cell viability and function were measured on days 7, 14 and 21.

### Optimisation of culture medium for 3D liver-bone co-culture system

To determine the optimal medium ratio that can support the viability and function of liver and bone cells, various ratios of HepaRG cell differentiation medium and bone differentiation medium (abbreviated in the text from now on as L-B medium or B-L medium) were tested for their effects on the viability and function of both compartments independently. 300 HepaRG spheroids or 2 bone scaffolds were cultivated in 100:0, 75:25, 50:50, and 25:75 ratios of L-B medium or B-L medium respectively. On days 7, 14, and 21, cell viability and function were measured compared to the control group of 100% HepaRG or bone differentiation medium respectively.

### Establishment of 3D liver-bone co-culture model

**Fig. 2:**
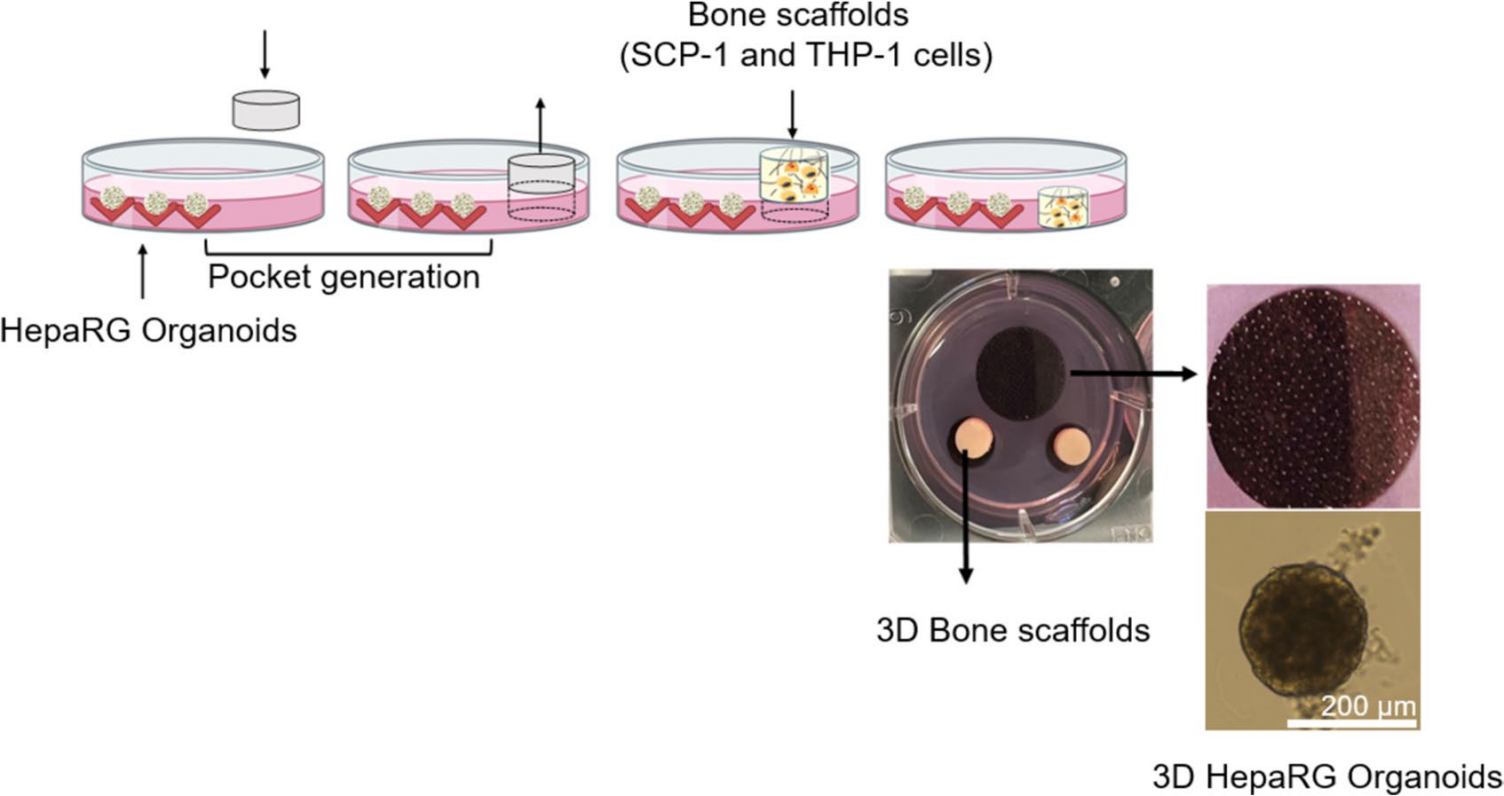
The setup of Liver spheroids-bone scaffolds co-culture system. After HepaRG spheroids were generated, the insert was used to make pockets for bone scaffolds on the agarose plate. Scaffolds containing bone cells were placed in the agarose pocket which combined with HepaRG spheroids to establish the 3D liver-bone co-culture model

Once the spheroids were generated, the pockets for the bone scaffolds were generated by piling the cell-free agarose. Scaffolds containing bone cells were placed in the agarose pocket (Fig. 2). The ratio between bone scaffolds and HepaRG spheroids was 2:300. After combining the scaffolds with the HepaRG spheroids, HepaRG cell differentiation medium and bone differentiation medium were used to maintain the co-culture system for 21 days. Cells were cultured in a humidified incubator at 37 °C with 5% CO_2_ and the medium was changed three times per week.

### Stimulation of diclofenac on the 3D liver-bone co-culture model

Diclofenac (D6899, Sigma) was dissolved in 99% formaldehyde to prepare a 10 mM stock solution, which was stored in a − 20 °C refrigerator. Based on clinical research findings, in vitro liver-bone model was treated with 3–6 µM diclofenac daily; those diclofenac concentrations represent the plasma concentration of diclofenac in humans after therapeutic use (oral intake 50 mg) (Cuklev et al. 2011; Miyatake et al. 2009; Scallion and Moore 2009).

### Stimulation of 4’-hydroxydiclofenac on the 3D bone co-culture model

4′-hydroxydiclofenac (32,412, Sigma; 4-OH diclofenac) was dissolved in 99% formaldehyde to prepare a 10 mM stock solution, which was stored in a − 20 °C refrigerator. Based on the amount of 4-OH diclofenac produced in our liver-bone model and the plasma concentration of 4-OH diclofenac after diclofenac use in humans. 75 nM, 300 nM, and 600 nM 4′-hydroxydiclofenac were choosed to exposure to 3D bone model daily (Degen et al. 1988; Yasar et al. 2001).

### Stimulation of Interleukin-6 on the 3D bone co-culture model

5 µg Interleukin-6 (200-06, Peprotech; IL-6) was first dissolved in 50 µl ddH_2_O, and then 450 µl 0.1% bovine serum albumin was added to prepare a 10 µg/ml stock solution, which was stored in a − 80 °C refrigerator. According to studies, 5–10 pg/ml IL-6 daily stimulation on a 3D bone model was used to mimic a human inflammatory condition. (Bakker et al. 2014; Singh et al. 2015).

### Mitochondrial activity assay by resazurin conversion

Mitochondrial activity for bone and liver cells were evaluated after the 7th, 14th and 21st days by resazurin conversion. Bone scaffolds were washed with PBS and transferred to a new 48-well plate. Next, scaffolds were immediately covered with 500 µL of a 0.0025% resazurin solution (in PBS). Scaffolds without cells were used as background controls. After incubation for 2 h at 37 °C, the fluorescence of the produced resorufin was measured using the Omega Plate Reader (BMG Labtech, Ortenberg, Germany) at excitation/emission (ex/em) 544 nm/590–10 nm. To measure the fluorescence, 3 × 100 µL of each sample (scaffold) was transferred to the wells of a 96-well plate (Haussling et al. 2019).

HepaRG spheroids were carefully collected from agarose microwells. Before measurement, the medium was aspirated and the spheroids were washed three times with HepaRG plain medium. 200 µL of the 0.0025% resazurin solution (in plain medium) was used for 300 HepaRG spheroids. 2 × 100 µL of HepaRG spheroids in resazurin working solution were transferred to a 96-well plate, as background 100 µL resazurin working solution without incubation with cells was used (McMillian et al. 2002). The produced resorufin was measured by the fluorescence at ex/em 544 nm/590–10 nm using the Omega Plate Reader after incubation for 30 min, 60 min, 90 min, and 120 min. Mitochondrial activity was calculated by the rate of conversion of resazurin to resorufin.

### CYPs activity assay by LC-HPLC/MS-based method (Phase I enzymes)

As described before (Hoffmann et al. 2012; Ruoss et al. 2019), CYP activities were measured based on the quantification of CYP-isoform-specific metabolites of different drugs using LC-HPLC/MS detection. Table 1 summarizes the selected substrates, working concentrations, incubation times, and the measured metabolites. 10 mM Phenacetin (77,440, Sigma), 10 mM Bupropion Hydrochloride (B689625, Toronto Research Chemicals), 10 mM diclofenac and 10 mM Testosterone (T1500, Sigma) stock solutions were diluted with HepaRG plain medium to a 1:1000 ratio and prepared as substrates for cocktail C1. 10 mM S-Mephenytoin (M225000, Toronto Research Chemicals) and 10 mM Bufuralol Hydrochloride (B689540, Toronto Research Chemicals) stock solutions were diluted with HepaRG plain medium to a 1:1000 ratio and prepared as substrates for cocktail C2. The HepaRG spheroids were incubated with 100 µL of working solution for C1 or C2 according to the described incubation time in Table 1. After incubation, the collected supernatants were stored at − 80 °C until measurement. Supernatants from 3 independent experiments were pooled into one sample and the CYP-isoform-specific metabolites of the sample were measured by the company Pharmacelsus (Saarbrücken, Germany) using an LC-HPLC/MS-based method. DNA content from liver spheroids was used to normalize data.

**Table 1:** CYPs activity by LC-HPLC/MS-based method (Phase I enzymes). Conditions, Substrates, concentrations, and measured reactions

Enzyme	Incubation time	(µM) Substrate → Metabolites
CYP1A2	1.5 h	(28 µM) Phenacetin → Phenacetin-deethylation
CYP3A4	1.5 h	(55 µM) Testosterone → Testosterone-6b-hydroxylation
CYP2B6	1.5 h	(111 µM) Bupropion → Bupropion-hydroxylation
CYP2C9	1.5 h	(10 µM) Diclofenac → Diclofenac-4′-hydroxylation
CYP2C19	3 h	(22 µM) S-Mephenytoin → Mephenytoin-4-hydroxylation
CYP2D6	3 h	(10 µM) Bufuralol Hydrochloride → Bufuralol-1-hydroxylation

### CYP2C9 activity assay by fluorescence-based methods (Phase I enzymes)

Phase I CYP2C9 enzyme activity was measured as previously described (Donato et al. 2015). Before measurement, the medium was aspirated, the HepaRG spheroids were washed three times with the HepaRG plain medium and 200 µL of the 5 µM Dibenzylfluoresceine and 10 µM 3,3′-Methylene-bis(4-hydroxycoumarin) (working solution—freshly prepared in HepaRG plain medium) were added to each sample of HepaRG spheroids. HepaRG spheroids in 100 µL with the working solution were transferred to a well of a 96-well plate in duplicates, and for the negative control, 100 µL of the working solution was transferred into a well without spheroids. The produced fluoresceine was measured by fluorescence at ex/em 485 nm/520 nm using the Omega Plate Reader after incubation for 30 min, 60 min, 90 min, and 120 min. CYP2C9 enzyme activity was calculated by the rate of Dibenzylfluoresceine to fluoresceine. DNA levels from liver spheroids were used to normalize data.

### UGT activity by fluorescence-based methods (Phase II enzymes)

An adjusted method previously reported was utilized to measure the activities of Phase II enzymes (Hammour et al. 2022). 200 µL of the 6.25 µM 4-Methylumbelliferone (in HepaRG plain medium) was used for 300 HepaRG spheroids. 100 µL HepaRG spheroids with the working solution were transferred to a 96-well plate to generate duplicates, and for the negative control, 100 µL of the working solution was transferred into a well without cells. The fluorescence of 4-Methylumbelliferone was measured at ex/em 355 nm/460 nm using the Omega Plate Reader after incubation for 30 min, 60 min, 90 min, and 120 min. DNA concentration from liver spheroids was used to normalize data.

### Alkaline phosphatase (AP) activity assay by absorbance-based method

As an initial indicator of osteogenesis, the activity of alkaline phosphatase (AP) was assessed (Aspera-Werz et al. 2018). The scaffolds were washed three times with PBS and then incubated with 500 µL of AP reaction buffer (3.5 mM 4-nitrophenyl-phosphate, 50 mM glycine, 1 mM MgCl_2,_ and 100 mM TRIS; pH 10.5) for a period of 2 h at 37 °C to allow for reaction. The photometric quantification of the conversion of 4-nitrophenyl-phosphate to 4-nitrophenol in 100 µL solution in triplicates was carried out (λ = 405 nm; Omega Plate Reader). The obtained experimental values were adjusted based on the background control, which was a scaffold without cells (Guo et al. 2022). Following this, the data was normalized to DNA concentration.

### Carbonic anhydrase (CA II) activity by absorbance-based methods

As an early indicator of osteoclast differentiation, CA II was assessed (Zhu et al. 2020). The cells/scaffolds were washed three times with PBS and then incubated with 100/500 µL of CA II reaction buffer (12.5 mM TRIS, 75 mM of NaCl, and 200 mM of 4-nitrophenyl acetate; pH 7.5) for 15 min at 37 °C. CAII activity was calculated by the rate of conversion of 4-nitrophenyl acetate to 4-nitrophenol. The resulting reaction’s product, 4-nitrophenol, was quantified using a photometer (λ = 405 nm; Omega Plate Reader). The obtained experimental values were adjusted based on the background control, which was a scaffold without cells (Guo et al. 2022). After that, the data was normalized to DNA concentration.

### Tartrate-resistant acid phosphatase (TRAP) activity by absorbance-based methods

A late marker for osteoclast activity, TRAP was assessed (Zhu et al. 2020). 30 µL of pooled supernatant was mixed with 90 µL of TRAP activity assay solution (5 mM 4-nitrophenyl phosphate, 100 mM sodium acetate, and 50 mM sodium tartrate in demineralized water; pH 5.5) to react for 6 h at 37 °C. To terminate the reaction, 90 µL/well of 1 M NaOH was added, effectively suppressing further reaction. The resulting reaction product, 4-nitrophenol, was quantified using a photometer (λ = 405 nm; Omega Plate Reader). The obtained experimental values were adjusted based on the background control, which was a scaffold without cells (Guo et al. 2022). After that, data was normalized to DNA concentration.

### Alizarin red staining

To assess matrix mineralization, cells were fixed with 100% ethanol overnight at − 20 °C. The fixed cells were gently washed with tap water, stained with 50 µL of 0.5% w/v Alizarin Red solution (pH 4.0), and incubated at room temperature for 30 min protected from light. Excess Alizarin Red staining solution was removed by washing the cells with tap water. For quantitative measurement, the bound Alizarin Red was dissolved with 10% w/v Cetylpyridinium chloride solution and quantified photometrically (λ = 562 nm; Omega plate reader) as described previously (Zhu et al. 2020).

### Sulforhodamine B (SRB) staining

SRB staining was performed to measure the total protein content of samples. Cells were directly fixed by ethanol for at least 60 min at − 20 °C after washing with PBS. Next, plates were incubated with 0.4% w/v SRB for 30 min at RT in the darkness. Then the samples were washed 4–5 times with 1% v/v acetic acid to remove unbound SRB. Afterwards, bound SRB was resolved with 10 mmol/L unbuffered TRIS solution (pH 10.5). SRB staining results were determined by absorbance (λ = 565 nm) with a plate reader (Ehnert et al. 2018).

### DNA isolation and quantification

As previously described (Haussling et al. 2019), DNA measurement was performed to assess cell viability and data normalisation. For the HepaRG spheroids, spheroids were collected from agarose microwell plates, washed with PBS, and then added to 100 µL of hot (98 °C) 50 mM NaOH solution and incubated at 98 °C for 5 min. After vortexing, the tubes containing the HepaRG spheroids and NaOH were frozen at − 20 °C. The following day, 110 μL TRIS (0.1 M, pH = 8.0) was added to all thawed samples and centrifuged at 20,000 × g for 10 min. For the bone scaffolds, scaffolds were transferred from agarose plates to a new 48-well plate. 250 µL of hot (98 °C) 50 mM NaOH solution was added to each scaffold. After 5 min of incubation, the scaffolds with NaOH were frozen at − 20 °C. The following day, 275 μL TRIS (0.1 M, pH = 8.0) was added to all thawed samples. After fully mixed, the liquid was collected and centrifuged at 20,000 × g for 10 min. For the absorption-based quantification, all samples were measured on the LVIS Plate (BMG Labtech, Ortenberg, Germany) in two steps. For the first step, 2 µL of DNA isolation buffer (500 µL NaOH (50 mM), and 550 µL TRIS (0.1 M, pH8.0)) as a blank was measured. In the second step, 2 µL of each DNA sample was measured in duplicates. Then, DNA concentrations were calculated at λ = 260 nm by the Omega analyzation software MARS.

### Mineral content of bone scaffold

The mineral content of the scaffold was analysed by performing quantitative computer tomographic (CT) scans utilizing a clinical CT scanner with 128 slice capability (SOMATOM Definition Edge, Siemens Healthineers, Erlangen, Germany). The specimens underwent scanning using the following parameters: an 80 kV tube voltage, an effective tube current of 500 mAs, an acquisition of 16 × 0.3 mm with a slice thickness of 0.4 mm, and a pitch of 0.4. Subsequently, the images were reconstructed using an advanced very sharp reconstruction kernel (V80u) with iterative image reconstruction technology (SAFIRE, Siemens Healthineers, Erlangen, Germany) with grade 5 and displayed in a bone window. Rectangular axial reconstruction field-of-view was about 10 cm x 10 cm with matrix resolution of 512 × 512 pixel. Obtained DICOM images were imported into the ImageJ software using the “DICOM sort” plugin. The resulting stack was cropped to show the area of interest. From each scaffold, the mean integrated density was determined and normalized to the reference block (Phantom EFP-06-96) (Weng et al. 2020).

### Stiffness of bone scaffold

The stiffness of the bone scaffold was measured using Young’s modulus as described previously (Weng et al. 2020). A ZwickiLine Z 2.5TN machine (Zwick GmbH and Co. KG, Ulm, Germany) vertically squeezed the surface scaffolds with the speed of 5 mm/min up to uniaxially by 10% of the original high of the scaffold. A force sensor recorded the applied force in real time. The stiffness of the scaffold was calculated:

$$Young^{\prime}s \;modulus\left[ {Mpa} \right] = \frac{{applied\;force \left[ N \right] \times initial\;scaffold \;height \left[ {mm} \right]}}{{area \;of\;the \;scaffold \left[ {mm^{2} } \right] \times change \;in \;height \left[ {mm} \right]}}$$

### Dot blot analysis

Dot blot was used to detect secreted protein in the culture supernatant, following the previously described method (Guo et al. 2022). 60 or 100 µL of supernatant was transferred onto a wet nitrocellulose membrane with a vacuum pump in a 96-well dot blotter (Carl Roth). After blocking them with 5% bovine serum albumin in TRIS-buffered saline/Tween-20 (TBS-T) for 1 h, membranes were incubated with primary antibodies at 4 °C overnight. After washing them with TBS-T, the membranes were incubated with the respective secondary antibody for 2 h. The antibodies used are summarized in Table 2. The signals were detected by chemiluminescence with a mixture containing 100 mM TRIS (T1503, Sigma), 1.25 mM Luminol (4203.1, Carl Roth), 0.2 mM p-coumaric acid (9908.1, Carl Roth), and 0.03 v/v H_2_O_2_ (CP26.5, Carl Roth), and quantified by ImageJ software.

**Table 2:** The antibodies used for dot blot

Antibodies	Order#	Company	Dilution
Osteocalcin (OCN)	sc-365797	Santa Cruz Biotechnology, Heidelberg, Germany	1:1000
Soluble receptor activator of nuclear factor kappa-B ligand (sRANKL)	500-m46	PeproTech, Hamburg, Germany	1:1000
Osteoprotegerin (OPG)	500-p149	PeproTech, Hamburg, Germany	1:1000
Interleukin-6 (IL-6)	500-m06	PeproTech, Hamburg, Germany	1:1000
Mouse anti-rabbit IgG-HRP	sc-2357	Santa Cruz Biotechnology, Heidelberg, Germany	1:10,000
Anti-Mouse IgG HRP-linked Antibody	7076	Cell Signaling Technology, Massachusetts, USA	1:10,000

### Detection of reactive oxygen species (ROS) by 2′,7′-dichlorofluorescein-diacetate (DCFH-DA) assay

HepaRG spheroids were washed three times with HepaRG plain medium before measurement. 100 µL of the 10 µM DCFH-DA working solution was used for HepaRG spheroids. After 30 min at 37 °C incubation, spheroids were washed by PBS three times. The spheroids were stimulated with diclofenac according to the setup of the experiment, and 0.03% H_2_O_2_ was used to stimulate spheroids as a positive control. The produced 2′7′-dichlorofluorescein (DCF) was continuously measured by the fluorescence at ex/em 485 nm/520 nm using the Omega Plate Reader during 0–10 min (Aspera-Werz et al. 2018).

### Detection of reduced glutathione (GSH) and oxidized glutathione (GSSG) by Ellman assay

After stimulation with 3–6 µM diclofenac, HepaRG spheroids were collected and washed three times with cold PBS before measurement. 3% W/V m-phosphoric acid was used to lysate cells to precipitate the proteins. Protein samples were re-neutralized with 5 mM EDTA in 0.1 M potassium phosphate buffer. After that, samples were centrifuged at 3000 × g for 10 min and then the supernatant was collected for GSH and total GSH measurements. For the determination of GSH, 20 µl of the sample was mixed with 120 µl of 0.56 mM 5,5′-dithiobis-(2-nitrobenzoic acid) (DTNB) in 0.1 M potassium phosphate buffer. To determine total GSH, 20 μl of the sample was incubated for 30 s with 120 μl of a mixture (1:1) of 1.68 mM DTNB and 2.5 U/ml glutathione reductase in 0.1 M potassium phosphate buffer. Then, 60 μl of NADPH 0.8 mM was added and absorbance was measured at λ = 412 nm. GSSG is obtained by subtracting GSH from the total GSH (Aspera-Werz et al. 2018).

### Statistical analyses

The data are presented as means ± the standard error of the mean (SEM). All the experiments were repeated at least three times with two to four technical replicates. Statistical analyses were performed using GraphPad Prism software (GraphPad Software 9.0, La Jolla, CA, USA). The data of the two groups were compared with the Mann–Whitney test. The data of multiple groups were compared with the non-parametric Kruskal–Wallis test, followed by Dunn’s multiple comparison test. A two-way ANOVA test followed by Turkey’s multiple comparisons was used when two independent variables were compared among groups. A *p-*value of < 0.05 was considered statistically significant.

## Results

### Impact of osteogenic factors contained in bone differentiation medium for HepaRG cells

**Fig. 3:**
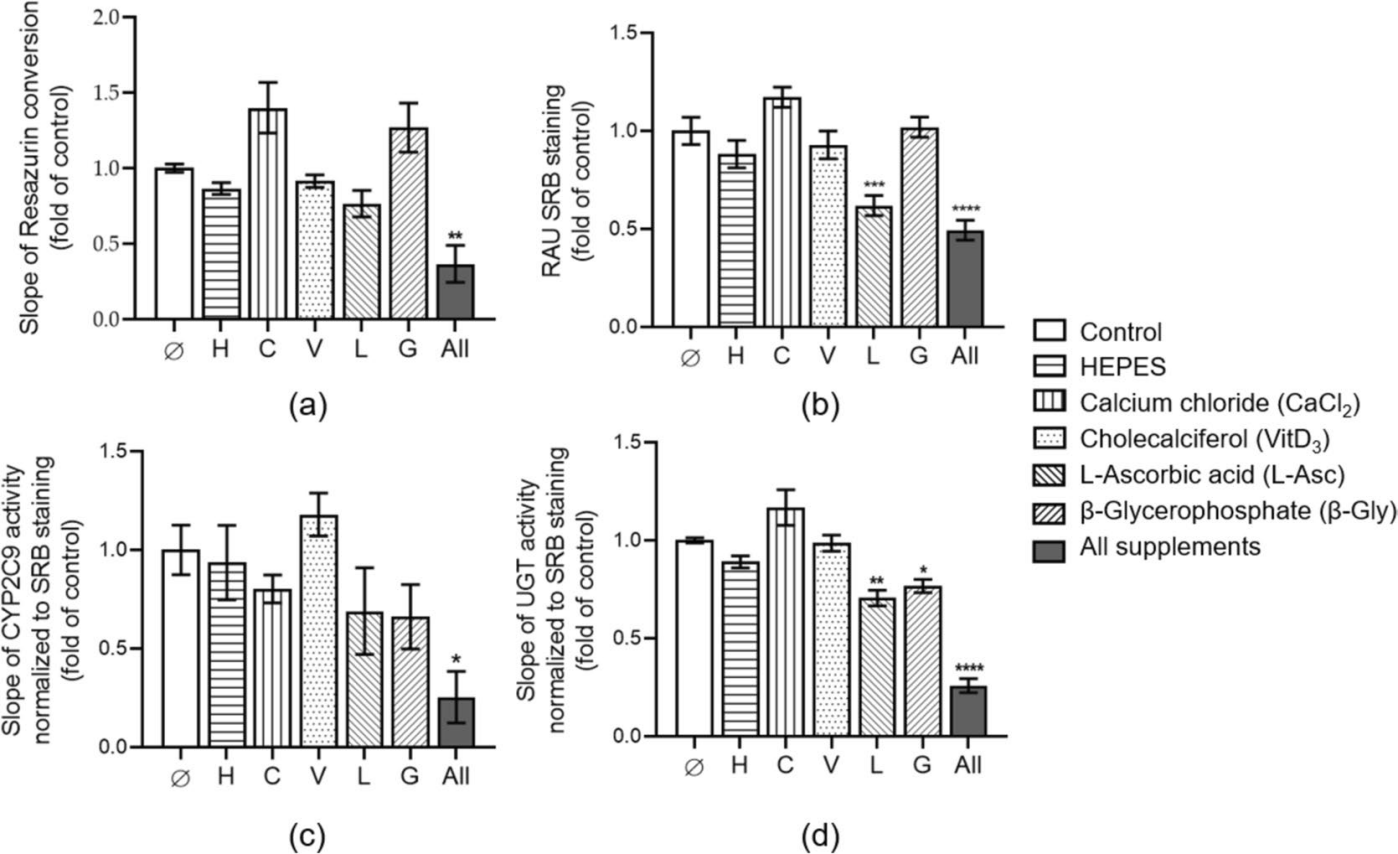
The effect of supplements contained in bone differentiation medium on HepaRG cells. HepaRG cells were separately or in combination incubated with HEPES [25 mM], Calcium chloride [1.5 mM], Cholecalciferol-VitD_3_ [20 ng/ml], L-Ascorbic acid 2-phosphate [200 µM] and β-Glycerophosphate [5 mM] for 21 days. **a** Resazurin conversion (mitochondrial activity) of HepaRG cells was measured on day 21. **b** The total protein content (SRB staining) of HepaRG cells was measured on day 21 and represented as relative absorbance units (RAU). For HepaRG cell function, the enzyme activity of Liver cells- CYP2C9 (**c**) and UGT (**d**) were compared with untreated cells on day 21. The Kruskal–Wallis test followed by Dunn’s multiple comparison test was used to determine statistical differences. Data are presented as means (fold of control) ± SEM, and the significance is shown as * *p* < 0.05, ** *p* < 0.01, *** *p* < 0,001, and **** *p* < 0.0001 vs. Control group. N = 3, n = 3

To evaluate the influence of several supplements, contained in the bone differentiation medium, HepaRG cells were stimulated with these factors: HEPES [25 mM], calcium chloride [1.5 mM], cholecalciferol [20 ng/ml], L-ascorbic acid 2-phosphate [200 µM] and β-glycerophosphate [5 mM]. After 21 days of exposure, HepaRG cell viability and function were tested. The mitochondrial activity of HepaRG cells was not significantly affected by any osteogenic factor tested alone; however, HepaRG cells exposed to a combination of supplements contained in bone differentiation medium showed a significant reduction in mitochondrial activity (*p* < 0.001; Fig. 3a). The tendency to increase the metabolic activity was observed in HepaRG cell cultures with calcium chloride or β-glycerophosphate. Total protein levels also confirmed the reduction of viable HepaRG cells in the group treated with the mixture of osteogenic components compared to control cells (*p* < 0.0001; Fig. 3b). To evaluate the influence of osteogenic factors on the drug metabolising activity of HepaRG cells, CYP2C9 activity was chosen as it is known that HepaRG cells’ CYP2C9 transcripts reach levels comparable to those of human hepatocytes (Aninat et al. 2006). Single exposure to supplements contained in bone differentiation medium did not influence CYP2C9 activity; nevertheless, exposure to the supplement mixture significantly downregulated CYP2C9 activity (*p* < 0.05; Fig. 3c). Likewise, UGT activity was reduced in the presence of mixed osteogenic supplements compared with untreated cells on day 21 (*p* < 0.0001; Fig. 3d). In addition, after 21 days of exposure, ascorbic acid 2-phosphate and β-glycerophosphate also significantly reduced UGT activity (*p* = 0.006 and *p* = 0.033; Fig. 3d).

### Impact of supplements contained in HepaRG cell differentiation medium on SCP-1 and THP-1 cell co-cultures

To assess the effect of HepaRG cell differentiation medium supplements on SCP-1 and THP-1 bone co-culture systems, 24 h after cell seeding (according to Sect. Bone cells co-culture), the system was stimulated with hydrocortisone [50 µM], L-glutamine [2 mM], DMSO [1.7%] and insulin [5 µg/ml] alone or in combination for 21 days.

**Fig. 4:**
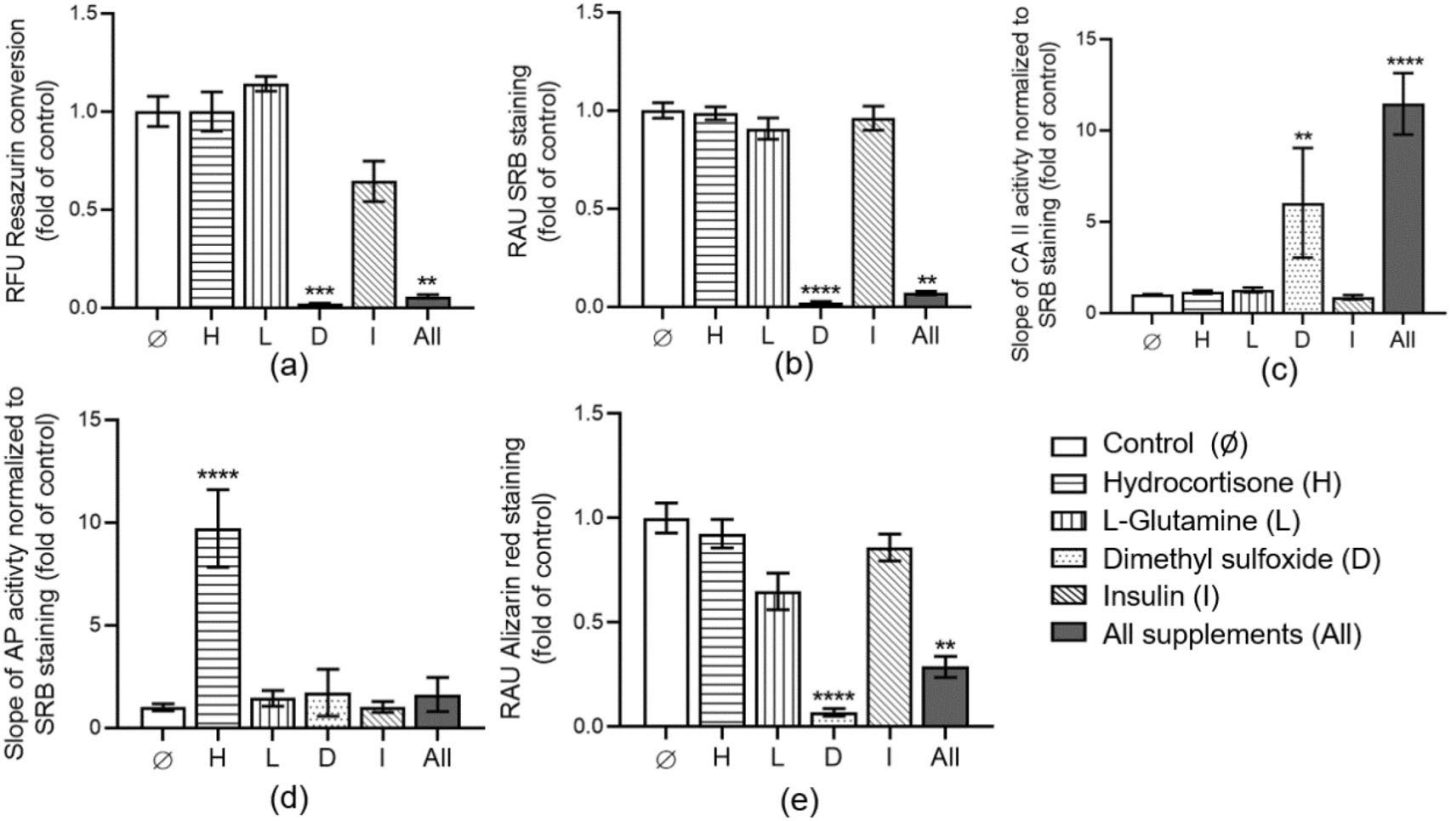
The effect of supplements contained in HepaRG cell differentiation medium on bone co-culture system. SCP-1 and THP-1 co-culture system was incubated separately or in combination with Hydrocortisone [50 µM], L-Glutamine [2 mM], DMSO [1.7%], and or Insulin [5 µg/ml] for 21 days. **a** Resazurin conversion (mitochondrial activity) of bone co-culture cells was measured on day 21 and represented as relative fluorescence units (RFU). **b** The total protein content of bone cells was measured on day 21 and represented as relative absorbance units (RAU). **c** CA II activity as the osteoclast function marker was measured on day 21. **d** AP activity as the osteoblast function was compared to untreated cells on day 21. **e** Total calcium staining of bone cells was measured on day 21 and represented as relative absorbance units (RAU). The Kruskal–Wallis test followed by Dunn’s multiple comparison test was used to determine statistical differences. Data are presented as means ± SEM, and the significance is shown as ** *p* < 0.01, ***p < 0.001 and **** *p* < 0.0001 vs. Control group. N = 3, n = 3

Our results showed that 1.7% DMSO and the combination of several supplements contained in HepaRG cell differentiation medium has a strong negative effect on bone cells’ mitochondrial activity (*p* = 0.0001, *p* = 0.0049; Fig. 4a) as well as total protein content (*p* < 0.0001, *p* = 0.0028; Fig. 4b) compared to the untreated bone cells. These results demonstrated the negative effect of DMSO on bone cell viability.

Regarding bone cell function, osteoblast-like cell function (analyzed by AP activity) was not negatively affected by any supplement tested. Interestingly, hydrocortisone significantly increased AP activity on the bone co-culture system (*p* < 0.0001; Fig. 4d). In addition, osteoclast-like cell function (determined by CAII activity) was increased with DMSO and all supplements mixed in the bone co-culture system, respectively (*p* = 0.0034, *p* < 0.0001; Fig. 4c). Total calcium staining also demonstrated an increase in bone-resorbing cell activity in the bone co-culture treated with DMSO and mixed supplements (*p* < 0.0001, *p* = 0.0011; Fig. 4e).

### HepaRG spheroids treated with different liver-bone medium mixes

**Fig. 5:**
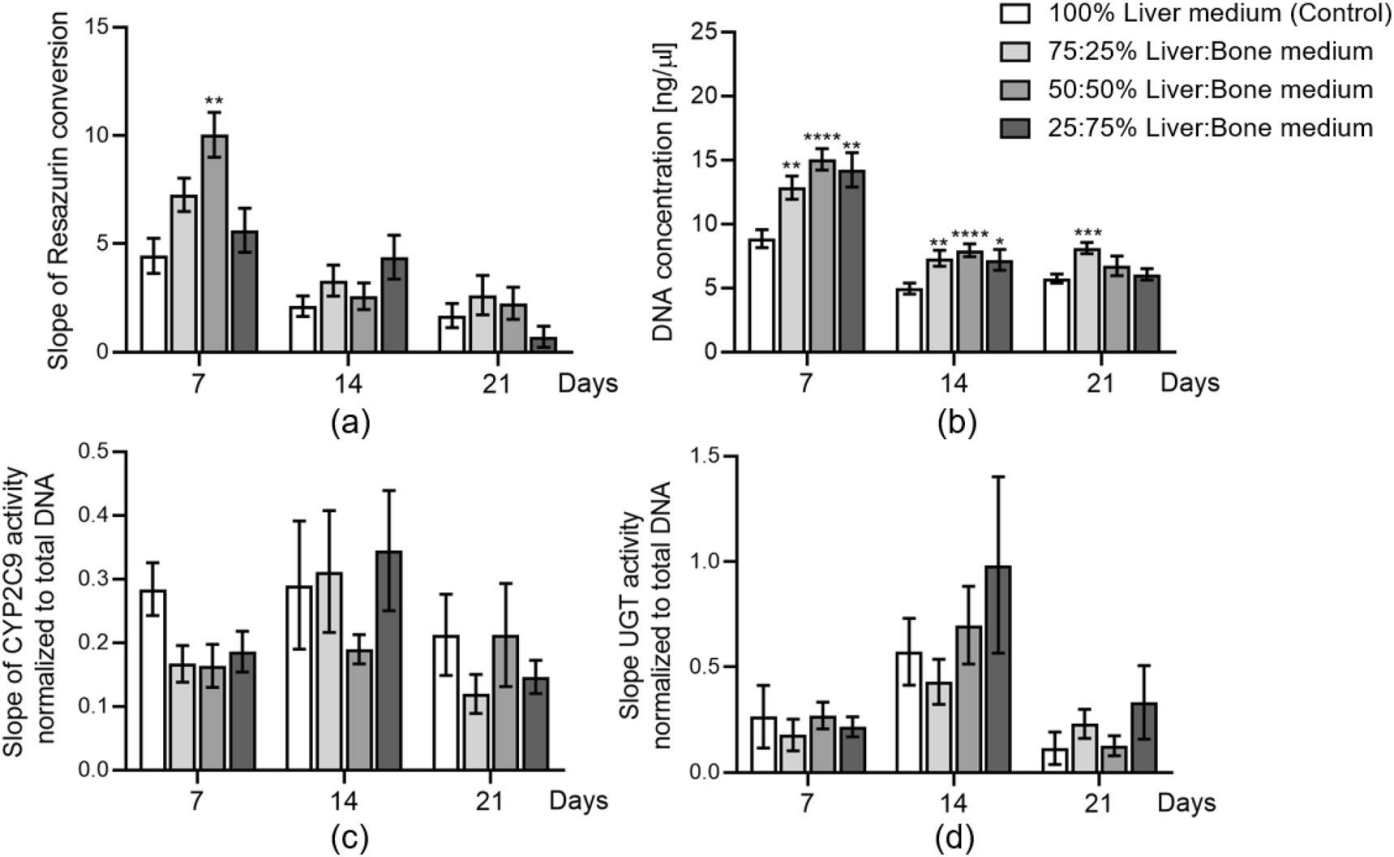
Medium test of liver-bone medium mixes on HepaRG spheroids. HepaRG spheroids were treated with liver-bone medium mixed 100:0, 75:25, 50:50, and 25:75 for 21 days. **a** Cell viability assessment by resazurin conversion (mitochondrial activity) on days 7, 14, and 21. **b** DNA content was measured to evaluate cell proliferation on days 7, 14, and 21. **c** CYP2C9 activity normalized to total DNA was measured to represent phase I enzyme activity on days 7, 14, and 21. **d** UGT activity normalized to total DNA as the marker of phase II enzyme activity was also measured on days 7, 14, and 21. The two-way ANOVA test followed by Turkey’s multiple comparisons test was used to determine statistical differences. Data are presented as means ± SEM, and the significance is shown as * *p* < 0.05, ** *p* < 0.01, *** *p* < 0,001, and **** *p* < 0.0001 vs. the Control group. N = 3, n ≥ 2

To determine the optimal cell culture medium that maintains viable and functional liver and bone cells for 21 days, HepaRG spheroids were grown in 100:0, 75:25, 50:50, and 25:75 ratios of HepaRG cells and bone co-culture differentiation medium (abbreviated in the text from now on as L-B medium). On days 7, 14, and 21, mitochondrial activity (Fig. 5a), DNA concentration (Fig. 5b), phase I enzyme CYP2C9 activity (Fig. 5c), and phase II enzyme UGT activity (Fig. 5d) were measured and compared to 100% HepaRG cell differentiation medium (control). The results of DNA content showed that the different ratios of L-B medium mixes increased the number of HepaRG cells after 7 and 14 days of culture compared to the control. However, at day 21, only 75:25 L-B medium significantly increased the DNA levels of HepaRG spheroids (Fig. 5b). Interestingly, mitochondrial activity showed only remarkable upregulation in the 50:50 L-B medium group on day 7 compared to the control (*p* = 0.005; Fig. 5a). Meanwhile, low mitochondrial activity was observed on HepaRG spheroids cultures with 25:75 L-B after 21 days relative to control HepaRG spheroids (0.709 ± 0.485; Fig. 5a). Regarding cell function, the different medium mixes tested did not have a negative effect on HepaRG spheroids’ function in terms of CYP2C9 activity and UGT activity (Fig. 5c–d). Based on these results, we concluded that all medium mixtures except 25:75 L-B medium support HepaRG spheroids functionality.

### Bone 3D co-culture system treated with different cell medium mixes

**Fig. 6:**
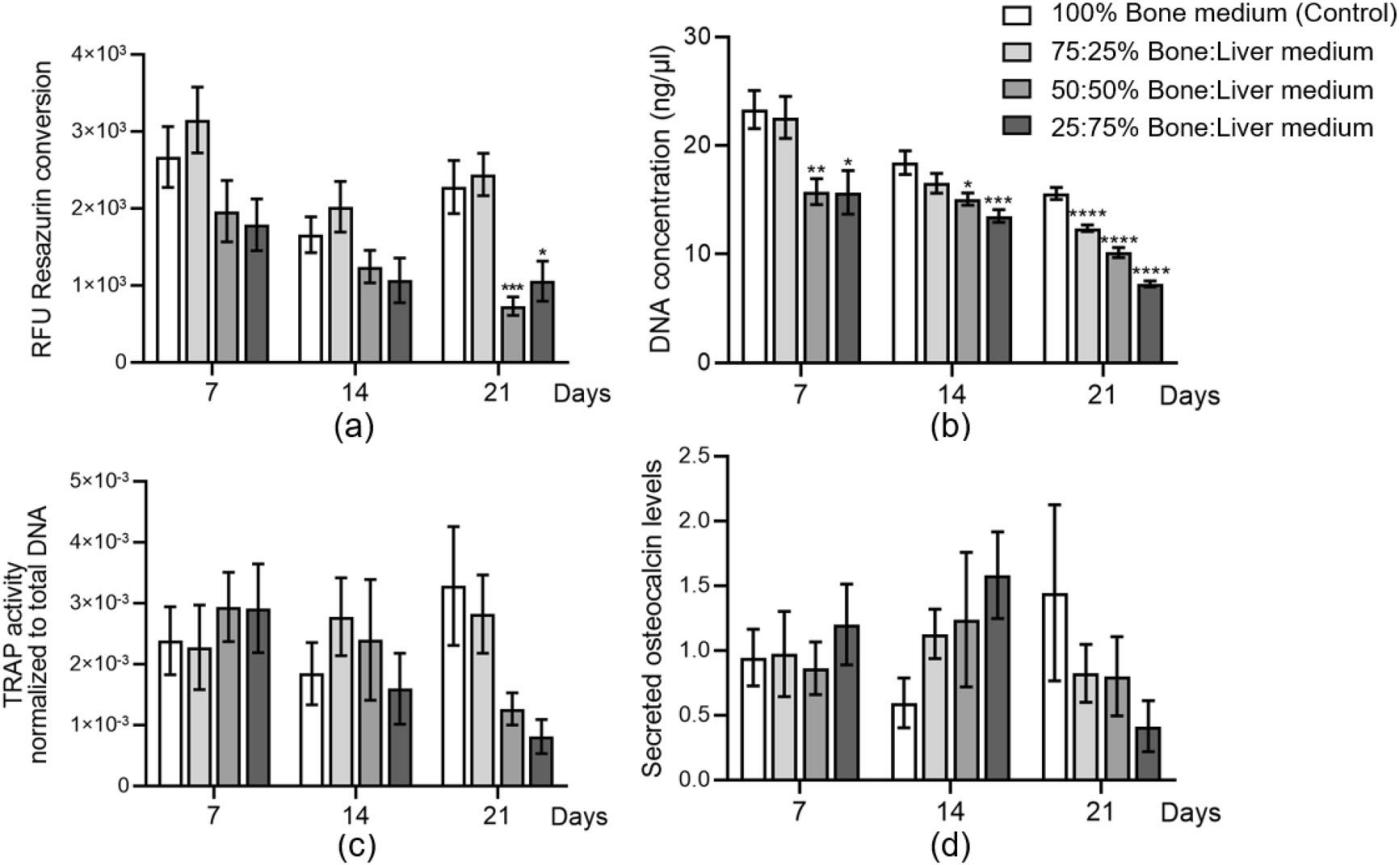
Medium test of bone-liver medium mixes on bone cells. Bone cells on scaffolds were treated with bone-liver medium mixed 100:0, 75:25, 50:50, and 25:75 for 21 days. **a** Cell viability assessment by resazurin conversion (mitochondrial activity) on days 7, 14, and 21 and represented as relative fluorescence units (RFU). **b** DNA content was measured to evaluate cell proliferation on days 7, 14, and 21. **c **TRAP activity normalized to total DNA was measured to represent osteoclast cell function on days 7, 14, and 21. **d** Secreted osteocalcin levels as the marker of osteoblast cells were also measured on days 7, 14, and 21. The Kruskal–Wallis test followed by Dunn’s multiple comparison test was used to determine statistical differences. Data are presented as means ± SEM, and the significance is shown as * *p* < 0.05, ** *p* < 0.01, *** *p* < 0,001, and **** *p* < 0.0001 vs. the Control group. N = 3, n ≥ 3

SCP-1 and THP-1 cells on PRP scaffolds were grown in 100:0, 75:25, 50:50, and 25:75 ratios of bone co-culture and HepaRG cell differentiation medium (abbreviated in the text from now on as B-L medium). Mitochondrial activity (Fig. 6a) and DNA concentration (Fig. 6b) were measured compared to 100% bone differentiation medium (control) to evaluate cell viability on days 7, 14, and 21. TRAP activity, a late-stage osteoclast differentiation marker, was measured to assess the function of osteoclast cells on days 7,14, and 21 (Fig. 6c). Osteocalcin, a non-collagenous protein formed during bone mineralisation, was detected as an osteoblast functional marker by dot blot on days 7, 14, and 21 (Fig. 6d). The results indicated that bone cell mitochondrial activity was markedly decreased in the 50:50 B-L and 25:75 B-L groups after 21 days of culture (*p* = 0.0006, *p* = 0.019; Fig. 6a). Bone cell exposure to the B-L medium mixture showed a significant dose- and time-dependent downregulation in DNA content relative to the control (Fig. 6b). Meanwhile, osteoclast TRAP activity showed a decrease in the 50:50 B-L and 25:75 B-L groups on day 21 compared to control (*p* = 0.207, *p* = 0.118; Fig. 6c). Additionally, the secreted osteocalcin level from osteoblast cells also decreased with the different ratios of B-L medium mixes on day 21 (*p* = 0.728, *p* = 0.728, *p* = 0.399; Fig. 6d). According to these results, all medium mixtures except 25:75 B-L medium maintained bone cell viability and functionality.

Considering the overall results in HepaRG spheroids and bone co-cultures, we concluded that a 50:50 medium mix is a suitable medium for the co-culture of HepaRG spheroids and bone scaffolds.

### HepaRG spheroids keep their viability and function up to 21 days in a liver-bone co-culture system

**Fig. 7:**
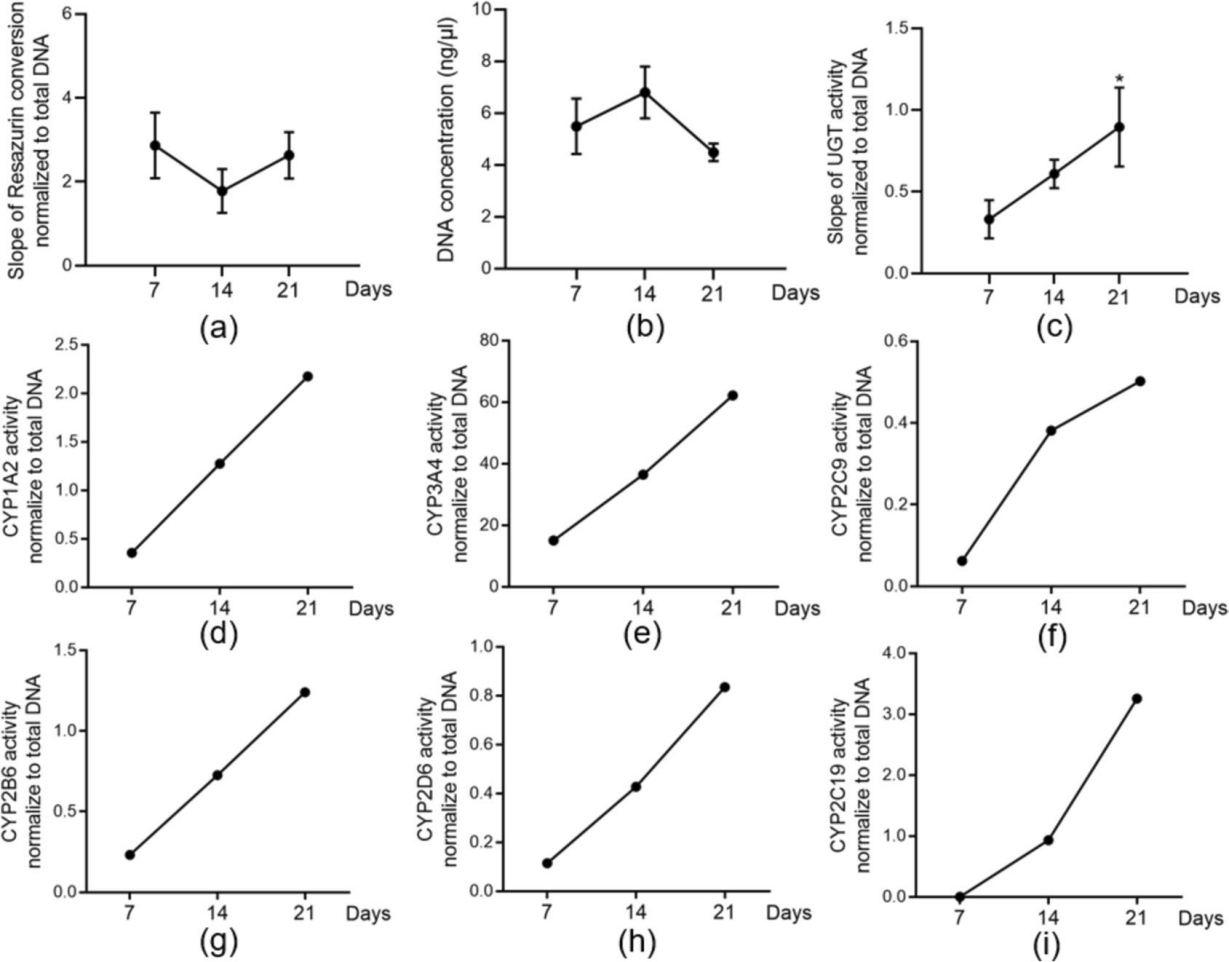
HepaRG spheroids viability and function after 21 days co-culture with 3D bone system. **a** Metabolic activity is evaluated by resazurin conversion (mitochondrial activity). **b** Cell viability assessment by DNA quantification. **c** UGT activity is normalized to the DNA content as the marker of phase II enzyme activity. The Kruskal–Wallis test followed by Dunn’s multiple comparison test was used to determine statistical differences. Data are presented as means ± SEM, and the significance is shown as * *p* < 0.05 vs. 7 Day group. N = 3, n ≥ 2. **d** CYP1A2 activity, **e** CYP3A4 activity, **f** CYP2C9 activity, **g** CYP2B6 activity, **h** CYP2D6 activity, and **i** CYP2C19 activity normalized to DNA content as the markers of phase I enzyme activity from 3 independent experiments pooled samples

Based on the results of the liver-bone medium optimisation (Sects. Bone 3D co-culture system treated with different cell medium mixes and HepaRG spheroids keep their viability and function up to 21 days in a liver-bone co-culture), a 50:50 liver-bone medium mixture is a suitable choice for keeping the model stable up to 21 days and it was used in further experiments. To evaluate HepaRG spheroids’ viability in the liver-bone co-culture system, mitochondrial activity, and total DNA content were measured on days 7, 14, and 21. Our results show no significant difference in mitochondrial activity and DNA content between 3-time points, which indicated that HepaRG spheroids were metabolically active and viable up to 21 days (Fig. 7a–b). As the most important function of drug detoxification, the cytochrome P450 (CYP) enzyme activities (CYP1A2, 3A4, 2B6, 2D6, 2C19, and 2C9) were measured with an LC-HPLC/MS-based method. The results showed that all CYP activity measurements showed a positive correlation in a time-dependent manner. (Fig. 7d–i). Moreover, the phase II enzyme UGT activity was also upregulated with time (Fig. 7c). These results demonstrated that HepaRG spheroids co-cultured with bone scaffolds are viable and showed drug metabolism capacity up to 21 days.

### The 3D bone system keeps its viability and function for up to 21 days in a liver-bone co-culture system

**Fig. 8:**
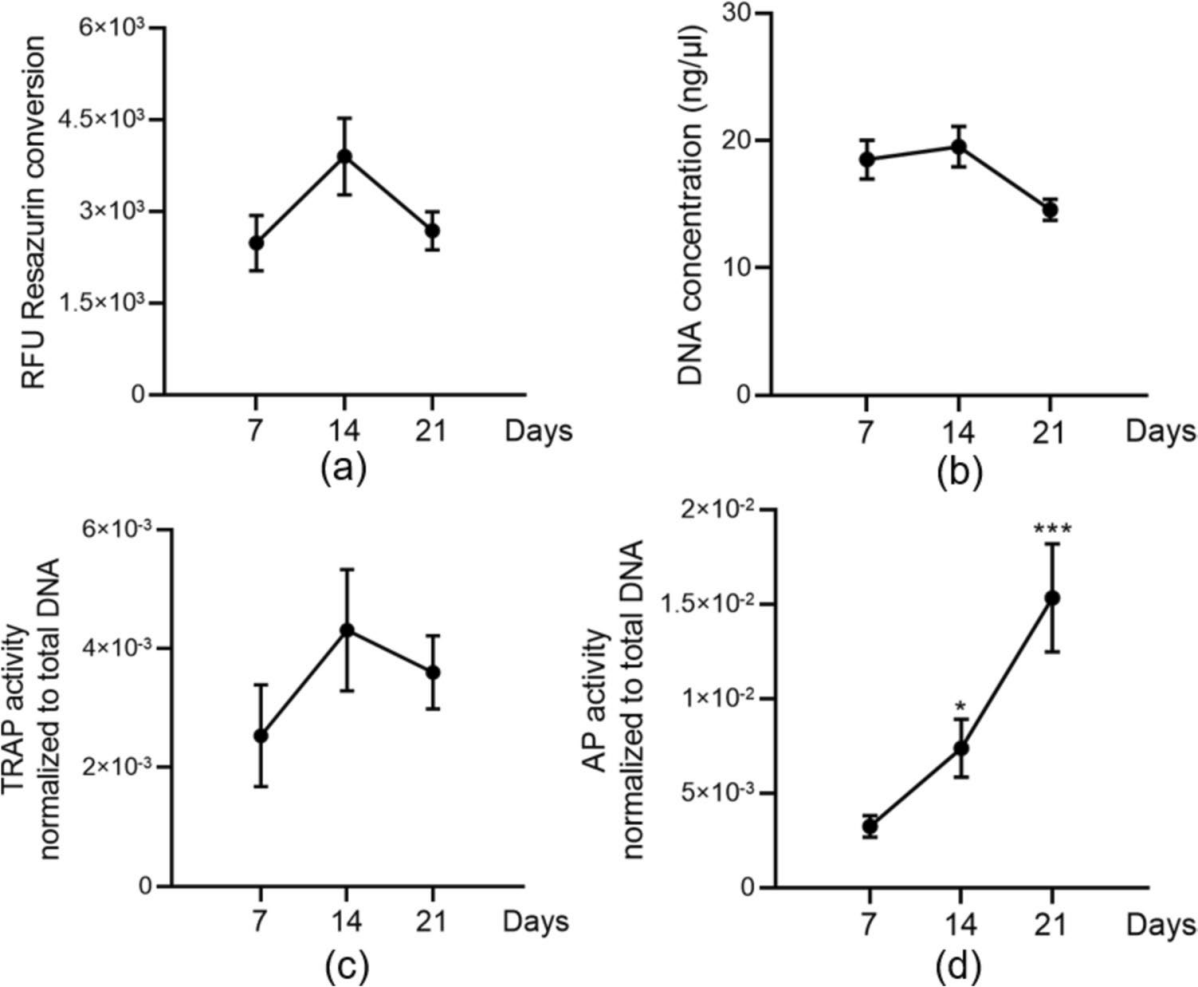
Bone co-culture system viability and function after 21 days of co-culture with HepaRG spheroids. **a** Bone cells metabolic activity was assessed by resazurin conversion (mitochondrial activity) and represented as relative fluorescence units (RFU). **b** Viability evaluated by DNA quantification. **c** TRAP activity as the marker of osteoclast function normalized to DNA content was measured. **d** AP activity as the marker of osteoblast function normalized to DNA content was measured. The Kruskal–Wallis test followed by Dunn’s multiple comparison test was used to determine statistical differences. Data are presented as means ± SEM, and the significance is shown as * *p* < 0.05 and *** *p* < 0,001 vs. the Day 7 group. N = 3, n ≥ 2

To evaluate the status of bone-forming/resorbing cell viability in the liver-bone co-culture system, mitochondrial activity and total DNA content were measured on days 7, 14, and 21. The results clearly showed that bone cell mitochondrial activity and total DNA were not negatively affected by co-culture with HepaRG spheroids (Fig. 8a–b), indicating that bone cells are viable in the liver-bone co-culture system for up to 21 days. The bone-resorbing cells’ activity (measured by TRAP activity) was upregulated after 14 and 21 days of co-culture with HepaRG spheroids compared to 7 days. However, there was no statistical significance between the three time points measured (Fig. 8c). Regarding bone-forming cell function, AP activity significantly increased in a time-dependent manner on the liver-bone co-culture system (*p* = 0.044, *p* = 0.0002; Fig. 8d). These results demonstrated that bone forming and resorbing cell activity was detected in the liver-bone co-culture system after 21 days.

### Chronic diclofenac exposure has no negative effect on HepaRG spheroids function in a liver-bone co-culture system

**Fig. 9:**
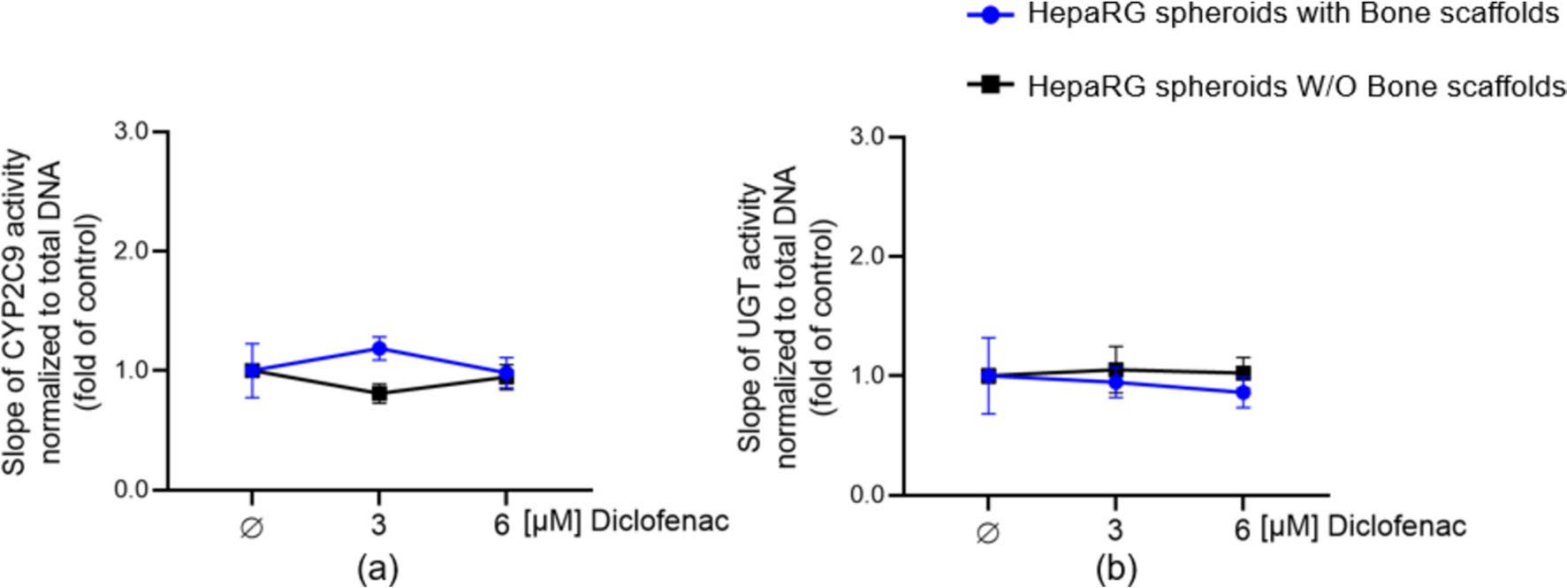
HepaRG spheroids and liver-bone system were stimulated with 3 and 6 µM diclofenac for up to 21 days. Diclofenac exposed to HepaRG spheroids with or without bone scaffolds, hepatocyte function assessment by CYP2C9 (**a**) and UGT (**b**) activity on day 21. The Kruskal–Wallis test followed by Dunn’s multiple comparison test was used to determine statistical differences. Data are presented as means ± SEM. N = 3, n = 2

Considering the overall results, we demonstrated that both compartments (HepaRG spheroids and bone scaffolds) preserve the viability and functionality of the target tissues over 21 days. Using this established liver-bone co-culture system, we decided to investigate the potential effects of diclofenac and its metabolites on bone cells. We daily exposed our liver-bone co-culture system to 3 or 6 µM diclofenac for 21 days. HepaRG spheroids and bone scaffolds alone (without co-culture) were also treated daily with 3 or 6 µM diclofenac (as a control). Diclofenac daily exposure showed no toxicity for HepaRG spheroids culture without or with bone scaffolds (Figure S1a–b). After daily exposure to diclofenac on days 7, 14, and 21, CYP2C9 and UGT activity (enzymes involved in diclofenac metabolism) were similar to those in the unstimulated condition (without diclofenac exposure) in HepaRG spheroids with and without bone scaffold co-culture (Fig. 9a–b). These results suggest that diclofenac metabolising capacity was not affected by diclofenac daily exposure in HepaRG spheroids co-cultures with bone scaffolds.

### Chronic diclofenac exposure increased the TRAP activity of osteoclasts in a liver-bone co-culture system

**Fig. 10:**
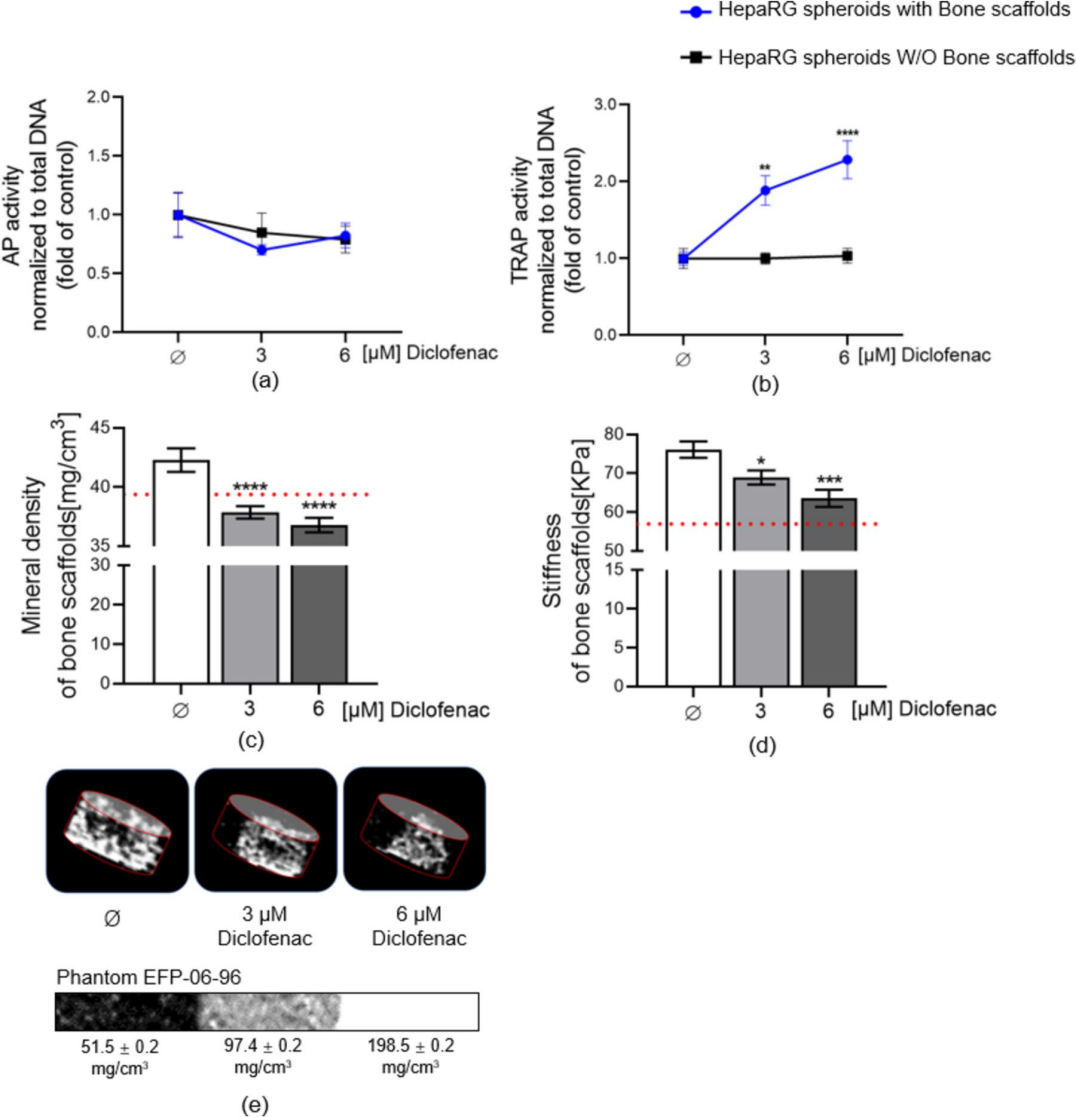
Bone and liver-bone systems were stimulated with 3 and 6 µM diclofenac for up to 21 days. Diclofenac was added to bone scaffolds with or without HepaRG spheroids, osteoblast function assessment by AP activity (**a**), and osteoclast function assessment by TRAP activity (**b**) on day 21. **c** Bone scaffold mineral content was measured by computer tomography on day 28 in the liver-bone system. The red line represents the mineral content of the scaffolds without cells. **d** Bone scaffold stiffness was measured by ZwickiLine machine on day 28 in the liver-bone system. The red line represents the stiffness of the scaffolds without cells. **e** Showing representative 3D reconstructions of the scaffolds by CT scan. The Kruskal–Wallis test followed by Dunn’s multiple comparison test was used to determine statistical differences. Data are presented as means ± SEM, and the significance is shown as * *p* < 0.05, ** *p* < 0.01, *** *p* < 0.001, and **** *p* < 0.0001 vs. the Control group. N = 3, n = 3

Regarding bone cells, diclofenac daily exposure did not show cytotoxicity for bone scaffold cultures with or without HepaRG spheroids (Figure S2a–b). In addition to assessing the effects on viability, the impact on osteoclast (TRAP activity) and osteoblast (AP activity) function markers were determined on bone cells treated daily with diclofenac in the liver-bone co-culture system. Our results clearly showed that bone scaffolds in liver-bone co-culture systems treated with diclofenac had comparable AP activity levels to bone scaffolds treated with diclofenac alone (Fig. 10a). However, TRAP activity was significantly upregulated after 21 days of exposure to diclofenac in bone scaffolds co-cultured with HepaRG spheroids (3 µM group *p* = 0.0036, 6 µM group *p* < 0.0001; Fig. 10b). In contrast, diclofenac did not increase TRAP activity in bone scaffold cultures without HepaRG spheroids (Fig. 10b). In addition to the increase in osteoclast activity, diclofenac significantly decreased the mineral content of the bone scaffolds co-cultured with HepaRG spheroids (*p* < 0.0001, *p* < 0.0001; Fig. 10c). These results suggest that diclofenac was metabolised by liver spheroids and then exerted its effects on bone-resorbing cells since this effect on TRAP activity was not observed when the bone scaffolds were not interacting with the functional liver compartment.

### 4′-hydroxy diclofenac (4-OH diclofenac) is not responsible for the upregulation of osteoclast cell function in the liver-bone system

**Fig. 11:**
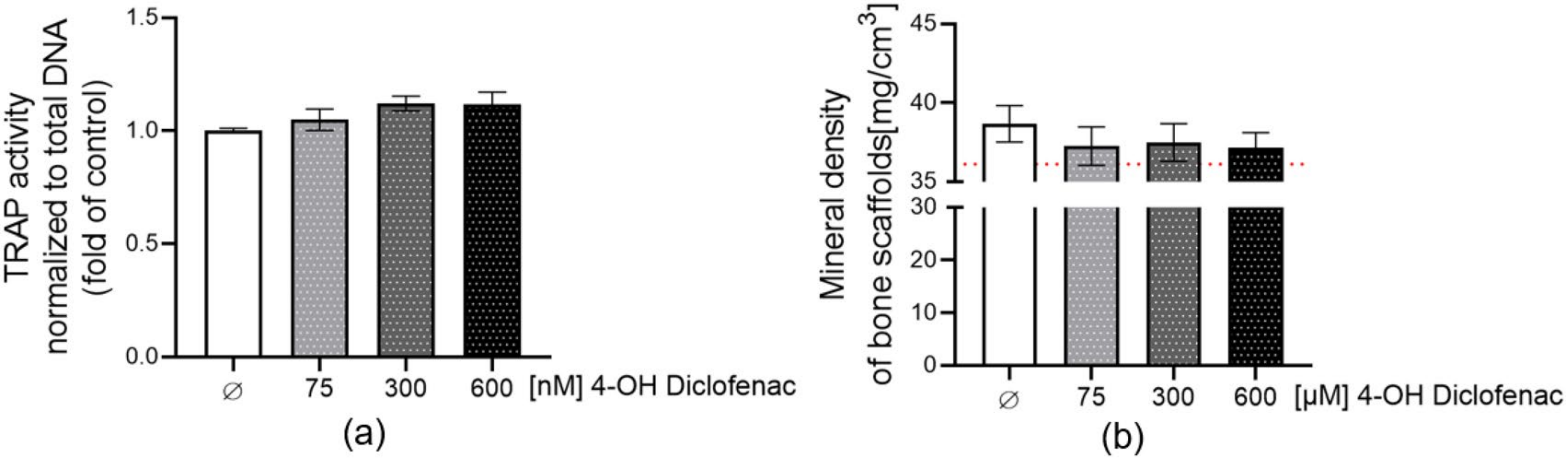
Bone co-culture systems were stimulated with 75, 300, and 600 nM 4-OH diclofenac for up to 28 days. **a** TRAP activity as the marker of osteoclast function normalized to DNA content was measured on day 21. **b** Bone scaffold mineral content was measured by computer tomography on day 28. The red line represents the mineral content of the scaffolds without cells. The Kruskal–Wallis test followed by Dunn’s multiple comparison test was used to determine statistical differences. Data are presented as means ± SEM. N = 3, n ≥ 2

4-OH diclofenac is the major metabolite of diclofenac produced by liver metabolism. To explore whether the major metabolite of diclofenac was responsible for the increased osteoclastic activity observed in our diclofenac-exposed liver bone system., we exposed the bone scaffolds to 4-OH diclofenac daily. 4-OH diclofenac did not show toxicity for bone cells after 21 days of exposure (Figure S3a–b). In addition, 4-OH diclofenac did not upregulate osteoclast cell activity after 21 days of exposure (Fig. 11a). The mineral density of scaffolds also did not change (Fig. 11b). These results demonstrated that 4-OH diclofenac did not affect the homeostasis of bone cells.

### Diclofenac exposure downregulates the GSH: GSSG ratio, and increases ROS and IL-6 levels in the liver system, causing the upregulation of RANKL and RANKL: OPG ratio in the liver-bone co-culture system

**Fig. 12:**
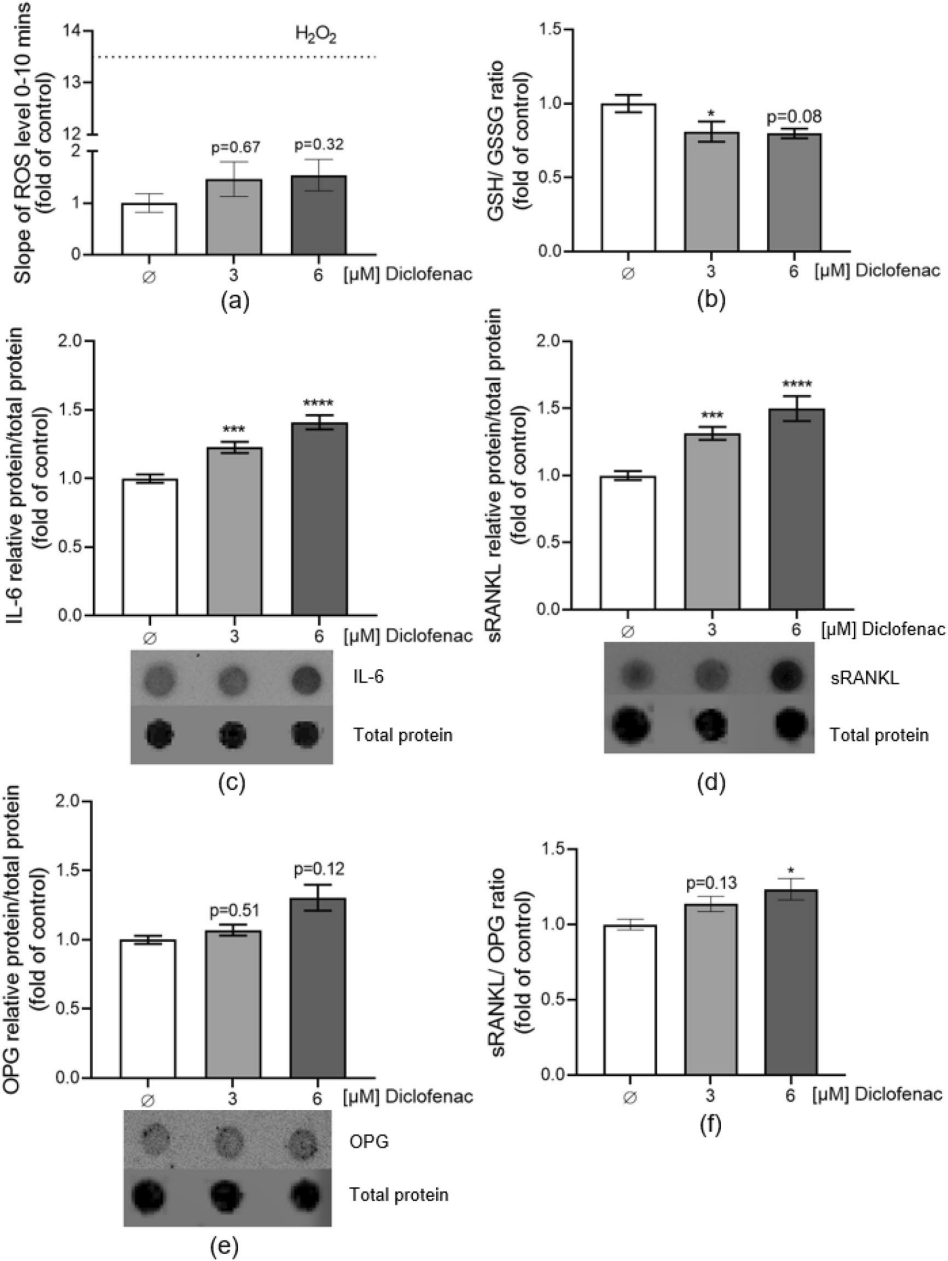
DCFH-DA assay was used to detect the ROS levels in liver spheroids. Ellman assay was used to detect the GSH and GSSG levels in liver spheroids. The expression of secreted IL-6, sRANKL, and OPG protein levels in the liver-bone system supernatant were measured by dot blot. **a** After acute exposure to diclofenac, the ROS levels in liver spheroids on day 7, H_2_O_2_ stimulation as a positive control. **b** After acute exposure to diclofenac, the ratio of GSH and GSSG in liver spheroids on day 7. **c** With the stimulation of 3–6 µM diclofenac, the IL-6 protein levels in supernatant on day 7, and the representative image of dot blot. **d** With the stimulation of 3–6 µM diclofenac, the sRANKL protein levels in supernatant on day 7, and the representative image of dot blot. **e** With the stimulation of 3–6 µM diclofenac, the OPG protein levels in supernatant on day 7, and the representative image of dot blot. **f** The ratio of sRANKL and OPG in the liver-bone co-culture system. The Kruskal–Wallis test followed by Dunn’s multiple comparison test was used to determine statistical differences. Data are presented as means ± SEM, and the significance is shown as * *p* < 0.05, *** *p* < 0.001, and **** *p* < 0.0001 vs. Control group. N ≥ 3, n ≥ 2

Diclofenac and its metabolites 4-OH diclofenac did not directly affect bone cells. Therefore, we hypothesize that inflammation and oxidative stress associated with chronic diclofenac intake are responsible for the increase in osteoclastic activity through the dysregulation of secreted cytokines. Single 3–6 µM diclofenac exposure was observed to elevate ROS levels within the liver system on day 7 (*p* = 0.387; Fig. 12a). Meanwhile, a decreasing level of GSH: GSSG was detected following a single exposure to 3–6 µM diclofenac in the liver system on day 7 (3 µM group *p* = 0.026, 6 µM group *p* = 0.08; Fig. 12b). Furthermore, daily diclofenac exposure significantly upregulates IL-6 (inflammatory mediator –secreted by hepatocytes) protein levels in supernatant in the liver-bone co-culture system on day 7 (3 µM group *p* = 0.0006, 6 µM group *p* < 0.0001; Fig. 12c). Elevated ROS and IL-6 are critical mediators of inflammatory responses and oxidative stress, which play pivotal roles in the regulation of bone metabolism. Afterward, the markedly increased secretion of RANKL (osteoclast activator) protein was detected in the diclofenac group by dot blot on day 7 (3 µM group *p* = 0.0001, 6 µM group *p* < 0.0001; Fig. 12d). Although OPG (RANKL decoy receptor) protein levels also increased, the rise was not as significant as that of RANKL (3 µM group *p* = 0.51, 6 µM group *p* = 0.12; Fig. 12e). Consequently, the ratio of RANKL: OPG increased in the diclofenac group compared to control (3 µM group *p* = 0.13, 6 µM group *p* < 0.05; Fig. 12f).

### IL-6 exposure upregulate the osteoclast cells function in the bone co-culture system

**Fig. 13:**
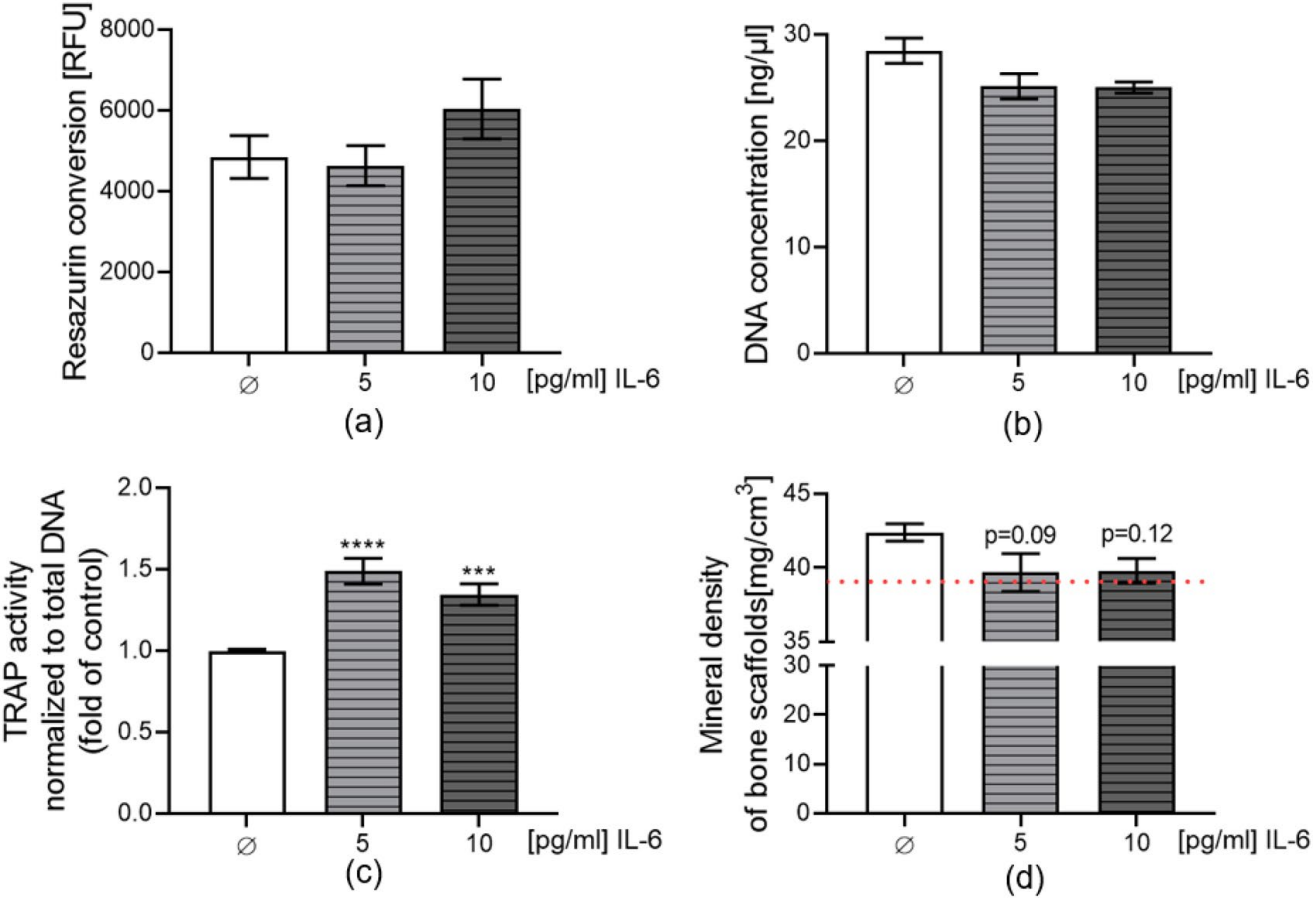
5 pg/ml and 10 pg/ml IL-6 exposure to 3D bone co-culture system for up to 21 days. **a** Bone cells metabolic activity was assessed by resazurin conversion (mitochondrial activity) and represented as relative fluorescence units (RFU). **b** Viability evaluated by DNA quantification. **c** TRAP activity as the marker of osteoclast function normalized to DNA content was measured. **d** Bone scaffold mineral content was measured by computer tomography scan on day 28. The red line represents the mineral content of the scaffolds without cells. The Kruskal–Wallis test followed by Dunn’s multiple comparison test was used to determine statistical differences. Data are presented as means ± SEM, and the significance is shown as ***p < 0.001 and ****p < 0.0001 vs. the Control group. N = 3, n ≥ 2

Our results showed elevated levels of IL-6 in the supernatant of the diclofenac group in the liver-bone co-culture system. To determine if this increased IL-6 affects bone homeostasis, we exposed the bone co-culture system to IL-6 daily. There are no significant changes in mitochondrial activity as well as in total DNA for bone cell viability after 21 days of IL-6 exposure (Fig. 13a–b). However, IL-6 significantly upregulated osteoclast cell activity after 21 days of exposure (5 pg/ml group *p* < 0.0001, 10 pg/ml group *p* = 0.0005; Fig. 13c). Meanwhile, IL-6 decreased the mineral content of the bone scaffolds on day 28 (5 pg/ml group *p* = 0.09, 10 pg/ml group *p* = 0.12; Fig. 13d). These results suggest prolonged exposure to high levels of IL-6 positively influences bone-resorbing cells.

## Discussion

The pathological mechanism of drug-induced osteoporosis is complex. Drugs and their metabolites can directly impact bone homeostasis, and they may also influence the bone by affecting liver function such as imbalances in hormone and vitamin D metabolism, as well as inflammation or immune responses, and eventually develop into hepatic osteodystrophy (HOD) (Ehnert et al. 2019). Drugs are metabolized in the liver and when the liver suffers damage from drug toxicity, it directly or indirectly affects bone homeostasis causing osteoporosis and increasing the risk of bone fragility. Several studies indicated that up to 75% of liver injury patients experience impaired bone homeostasis (Angulo et al. 2011; Ehnert et al. 2020; Sriuttha et al. 2018). Currently, there have been some animal model-based studies to investigate and understand the mechanisms underlying HOD (Sens et al. 2017; Spirlandeli et al. 2017). However, animal models have certain limitations to consider. Apart from being costly, time-consuming, and ethically challenging, they may also be difficult to replicate, especially under complex experimental conditions. The most significant flaw in animal models is their potential inability to accurately reflect human biology or diseases and the results of animal experiments may not directly apply to humans (Aerssens et al. 1998; Ehnert et al. 2020). The interspecies variations between humans and animals regarding drug metabolism, tolerance of drug toxicity, and bone strength highlight the need for a human in vitro liver-bone model. 2D cell models are unable to accurately replicate the complex three-dimensional structures and cell interactions that are present in living tissues, limiting their predictive capabilities. Furthermore, two-dimensional cell models may not accurately predict in vivo drug efficacy or toxicity, which can restrict their applications in drug discovery and development (Schyschka et al. 2013). Therefore, to better mimic the in vivo scenario, screen toxicity of drugs on bone homeostasis, and understand the mechanisms responsible for HOD in humans, it is necessary to develop a long-term stable in vitro 3D liver-bone co-culture model that represents the relevant interactions between the two tissues.

So far there has been no liver–bone in vitro co-culture model that represents the interaction between both organs. As previously described, due to pore size and stiffness, hPRP scaffolds are a suitable platform with which to cultivate osteoblast-osteoclast-like cells. Additionally, changes in scaffolds’ mineral content were detected. Therefore, SCP-1 and THP-1 cell lines on hPRP scaffolds were chosen to represent the bone tissue (Haussling et al. 2019; Weng et al. 2020). For the liver model, due to the remarkably higher CYP activity compared to that of other common human hepatic cell lines (*e.g.* HepG2, Huh-7), which was similar to the levels of freshly isolated human hepatocytes, HepaRG spheroids were chosen to represent liver tissue (Dean and Kane 2012; Lin et al. 2012). Our goal was to establish a new in vitro 3D co-culture model of bone and liver-like cells mimicking the human situation and providing a tool to screen drug toxicity and study the pathomechanism(s) of possible interactions of the liver-bone axis.

Since the differentiation medium for liver cells and bone cells are different, the supplements contained in the medium may exert detrimental effects on other cell types. Therefore, as a first step, we evaluated the impact of the supplements in the differentiation culture medium on the viability and functionality of liver and bone cells. Our results showed that 200 µM L-ascorbic acid 2-phosphate negatively affected HepaRG cell proliferation and function. However, a previous study demonstrated that 200 µM L-ascorbic acid has beneficial effects on mesenchymal cell proliferation (Mekala et al. 2013). This dual role could be associated with the antioxidant and pro-oxidant behaviour of L-ascorbic acid (Dhage and Sharbidre 2022). In addition, our results showed that 50 µM hydrocortisone significantly upregulated AP activity in bone cells. This result was supported by the studies of Griffin et al*.* and Bazzell et al*.*, which reported an increased AP activity in HeLa cells treated with hydrocortisone or cortisol (Bazzell et al. 1976; Griffin and Ber 1969). However, Bazzel et al*.* demonstrated that this increase in AP enzyme activity is due to the enhanced catalytic efficiency of cortisol rather than due to an increase in enzyme protein expression levels (Bazzell et al. 1976). As a result, we can hypothesise that the increase in AP observed in bone cells exposed to hydrocortisone is not associated with an increase in bone-forming cell differentiation. Furthermore, hepatic factors had a negative effect on the bone co-culture system, particularly 1.7% DMSO, which strongly and negatively affected cell viability and matrix mineralisation. Interestingly, increased CAII activity was observed in bone co-culture cells treated with 1.7% DMSO. However, some researchers have reported that DMSO inhibited the differentiation and function of osteoclast cells (Lemieux et al. 2011; Yang et al. 2015). Since our data were normalized to total bone cells (including osteoblasts and osteoclasts), this increase in CAII activity could be due to the effects of 1.7% DMSO on bone cell viability. Although some studies have shown that HepaRG could be differentiated without DMSO (Rose et al. 2022), to maintain hepatic function for a long period, we chose to use a lower concentration of DMSO (0.85%) in our liver-bone co-culture system.

We attempted to find an appropriate differentiation medium for cultivating the liver-bone system. In the next step, we tested different ratios of liver-bone differentiation medium to assess their effects on the 3D liver and bone model separately. For the liver assay, the results of resazurin conversion showed less mitochondrial activity in the 25:75 L-B medium group compared to the control after 21 days. Meanwhile, in terms of DNA content, the 25:75 L-B medium did not significantly decrease compared with other groups. This means that the 25:75 L-B medium group negatively affected HepaRG spheroids’ metabolic activity. For the bone co-culture system, 75:25 L-B medium showed lower TRAP activity and OC levels compared to 50:50 L-B medium. Since our results demonstrated that 1.7% DMSO contained in L medium had a negative effect on bone cells, we decided to use a medium mixture containing less DMSO to protect the viability and function of the bone cells without affecting HepaRG spheroids differentiation. All in all, to maintain the viability and functionality of liver and bone cells and preserve them to the maximum extent for 21 days, we chose the 50:50 liver-bone mixed differentiation medium for culturing the liver-bone cell model.

Diclofenac is a commonly used NSAID that exerts analgesic and anti-inflammatory effects by inhibiting the conversion of arachidonic acid to prostaglandins (Gunaydin and Bilge 2018). It is frequently used in various orthopaedic conditions involving mild to moderate acute or chronic inflammation (Al-Waeli et al. 2021; Altman et al. 2015; Ku et al. 1986). Upon entering the body, because of pre-systemic metabolism, 40% of diclofenac undergoes metabolism in the liver by hepatocytes CYP2C9, CYP3A4, and UGT enzymes to form 4-OH diclofenac and 5-OH diclofenac, which then with the remnant diclofenac circulate through the bloodstream to exert its effects in various parts of the body (Dean and Kane 2012; Tateishi et al. 2020; Todd and Sorkin 1988). However, in vivo animal reports suggest that diclofenac can affect bone homeostasis and impair fracture healing (Cottrell and O’Connor 2010; Karanikola et al. 2022; Menger et al. 2023). Despite the direct effects that diclofenac may have on bone cells, we should also consider the indirect role of diclofenac on bone cell homeostasis caused by liver damage. Therefore, the changes in cytokine production by hepatocytes following diclofenac exposure, such as bone morphogenetic protein 9, insulin-like growth factor 1, transforming growth factor beta (TGF-β), sclerostin, interleukin 6 and interleukin 11, could affect bone homeostasis (Ehnert et al. 2019). However, the precise mechanisms underlying the impact of diclofenac on bone homeostasis remain unclear, as most in vitro studies on bone either exclude the liver, which is essential for drug metabolism or rely on animal models (Karanikola et al. 2022; Menger et al. 2023), lacking an in vitro human liver-bone cell co-culture model for investigation.

In our study, we aimed to use diclofenac to validate the functionality of our liver-bone co-culture system, and to investigate the toxicity of diclofenac on bone homeostasis. Our results showed that diclofenac exposure, either to hepatocyte spheroids alone or within the liver-bone co-culture model, did not significantly impact the basal enzymatic function of CYP2C9 and UGT (two important enzymes responsible for metabolising diclofenac (Knospel et al. 2016)). This suggests that the hepatocytes in our co-culture model are functional and capable of metabolising diclofenac continuously. When diclofenac was exposed solely to the 3D bone cell model, the functionality of osteoclasts and osteoblasts was not significantly affected. However, interestingly, when diclofenac was added to the liver-bone co-culture model, we observed a significant enhancement of the functionality of osteoclasts (bone-resorbing cells) in the diclofenac group on day 21. Also, after 28 days of culture, we performed a CT scan and stiffness test of our bone scaffold co-culture with liver spheroids and found a significant decrease in mineral density and stiffness in the diclofenac group. Our results are in line with previous preclinical and clinical studies that demonstrated a decrease in bone mineral density after diclofenac exposure in animal models and orthopaedic patients (Cottrell and O’Connor 2010; Dodwell et al. 2010; Gaston and Simpson 2007; Hatipoglu et al. 2015; Hernandez et al. 2012; Krischak et al. 2007).

It was reported that the diclofenac half-life is approximately 2 h, however, the half-life including all metabolites is 25.8–33 h (Todd and Sorkin 1988). Therefore, we propose that the primary metabolite of diclofenac, 4-OH diclofenac (Bouju et al. 2016), may enhance osteoclast differentiation and function, thereby affecting bone cell homeostasis. However, our results showed that 4-OH diclofenac does not upregulate osteoclast function when applied directly to the bone co-culture system. Our experimental results showed that neither diclofenac nor its metabolite 4-OH diclofenac is responsible for the increased osteoclast activity observed in the liver-bone in vitro system. Hence we hypothesized that the changes in hepatocyte-secreted factors induced by diclofenac-associated oxidative stress and inflammation may enhance osteoclast differentiation and function.

When drugs enter the liver, they typically undergo a series of metabolic processes to be eliminated from the body. This process is primarily catalyzed by the liver’s enzyme systems, such as the cytochrome P450 family of enzymes (Zhao et al. 2021). This metabolic process is often accompanied by the generation of ROS, especially when the drug or its metabolites have the potential to induce oxidative stress (Allameh et al. 2023). In the liver, GSH plays a crucial antioxidant role, which can directly react with ROS through its thiol group, converting ROS into harmless substances. During this reaction, GSH could be oxidized to GSSG, the ratio of GSH and GSSG normally used to assess the antioxidant ability of cells (Vairetti et al. 2021). When the generation of ROS exceeds the cell’s antioxidant defense capacity, the cell enters a state of oxidative stress (Lee et al. 2022). Under oxidative stress, inflammatory factors are released into the extracellular space, triggering an inflammatory response. These inflammatory factors not only affect liver cells but can also impact bone cell function through the liver-bone axis. In agreement with others in vivo and in vitro studies (Ahmad et al. 2013; Alabi et al. 2017; Thai et al. 2023), our results show that diclofenac exposure increases oxidative stress and reduces total GSH. As GSH neutralizes free radicals and conjugates with diclofenac to protect cells from oxidative stress and facilitate diclofenac detoxification (Sousa et al. 2021), lower total GSH leads to liver cell damage following diclofenac chronic intake (Mechcatie 2010).

IL-6 is a multifunctional cytokine that plays a crucial role in bone metabolism. It is reported that IL-6 binds to its receptor (IL-6R), and this complex directly interacts with the signal transducer molecule gp130 on osteoclast precursors, promoting their differentiation into mature osteoclasts (Feng et al. 2017; Mihara et al. 2012). Apart from the direct influence, IL-6 can also act on osteoblasts, promoting the secretion of RANKL by activating the JAK/STAT3 pathway, thus facilitating the differentiation and activation of osteoclasts (Harmer et al. 2019; Wu et al. 2017). This is consistent with our experimental results, as RANKL levels increased in the supernatant. OPG functions as a decoy receptor for RANKL, effectively competing with RANK on osteoclast precursor cells (Lacey et al. 1998). In our results, the OPG also showed an increased trend in the supernatant, and we postulate that OPG may rise compensatorily as a regulatory mechanism. The final ratio of RANKL to OPG is increased, which is beneficial for osteoclast differentiation and function (Boyce and Xing 2008; Udagawa et al. 2021). We determined the dosage of IL-6 based on its reported concentration in human serum. Generally, the serum concentration of IL-6 in healthy individuals is less than 5 pg/ml (Singh et al. 2015). We exposed our bone co-culture system to 5 pg/ml and 10 pg/ml of IL-6 to simulate the cytokine concentration under inflammatory conditions in the human body. We found that IL-6 indeed increased the TRAP activity of osteoclast cells.

In addition to drug toxicity testing, our system has the potential to study the interactions between these organs on the liver-bone axis. The liver is not only the organ responsible for the detoxification of drugs but also produces many factors, that influence bone homeostasis (*e.g.* bone morphogenetic protein 9, insulin-like growth factor 1, TGF-β, interleukin 11, vitamin D metabolism and parathyroid hormone metabolism (Ehnert et al. 2019)). Therefore, it is expected that a malfunction of the liver positively correlates with impaired bone homeostasis, and the associated molecular mechanisms can be studied with the liver-bone in vitro system. For instance, liver fibrosis is associated with increased levels of TGF-β1 (Fabregat et al. 2016), and various inflammatory cytokines, as well as decreased production of insulin-like growth factor (Liu et al. 2018), lecithin-cholesterol acyltransferase (Lu et al. 2022), and alterations in vitamin D and parathyroid hormone metabolism. These factors play important roles in maintaining bone homeostasis, and their dysregulation can have direct or indirect effects on bone health. By combining HepaRG cells with TGF-β1-activated LX-2 cells (as stellate cells), we have established a fibrosis model (Zahmatkesh et al. 2022) that, when cultured with bone scaffolds, can contribute to the study of the relationship between fibrotic liver and bone.

When liver cells are subjected to oxidative stress, besides IL-6, many other cytokines such as tumor necrosis factor, TGF-β, and other interleukin family members may also undergo changes. Therefore, those cytokines can also negatively impact the homeostasis of bone cells, needing further research. This study also has some limitations. Due to the constraints of cellular experiments, it is challenging to fully replicate the most realistic conditions within the human body. Maintaining the homeostasis of bone tissue involves the influence of various cell types, including endothelial cells, immune cells, bone lining cells, osteocytes, and chondrocytes, which are not represented in our model. Additionally, the liver part of our study is limited to hepatocytes and cholangiocytes; however, other cell types (*e.g.*, Kupffer cells, hepatic stellate cells, and endothelial cells) may also play an important role in liver tissue damage and influence the function of hepatocytes. Further research should consider the incorporation of Kuppfer cells into the liver system to better represent the inflammation response associated with drug-induced liver injury.

## Conclusion

We here established for the first time an in vitro liver-bone co-culture system consisting of human hepatoma-derived cell (HepaRG) spheroids co-cultured with scaffolds containing a human MSC line (SCP-1) and a human monocytic cell line (THP-1) co-cultured on an agarose platform. We tested the response of our liver-bone co-culture system to daily pharmacology diclofenac concentration and observed that bone homeostasis was disrupted in our liver-bone system after diclofenac exposure due to increased bone resorption activity. We propose that chronic diclofenac intake may induce an inflammatory response in hepatocytes via oxidative stress, thereby influencing bone homeostasis and delaying bone fracture healing. These results demonstrated that diclofenac was properly metabolised by liver spheroids and then exerted its negative effects on bone cells. We emphasized that the liver system plays an important role in screening the toxicity of drugs on bone homeostasis. The 3D liver-bone system could be a promising model for studying the toxicity of drugs on bone homeostasis and representing physiologically impaired bone metabolism under liver damage.

## Supplementary Information

Below is the link to the electronic supplementary material.

Supplementary file1 (DOCX 200 KB)

## Data Availability

The datasets and/or analyzed generated during the current study are available from corresponding author on reasonable request.

## References

[CR1] Adnan Memic TC, Eggermont LJ, Rezaeeyazdi M, Joseph Steingold ZJR, Navare KJ, Mohammed HS, Bencherif SA (2019) Latest advances in cryogel technology for biomedical applications. Adv Therap 2(4):1800114. 10.1002/adtp.201800114

[CR2] Aerssens J, Boonen S, Lowet G, Dequeker J (1998) Interspecies differences in bone composition, density, and quality: potential implications for in vivo bone research. Endocrinology 139(2):663–670. 10.1210/endo.139.2.5751 9449639 10.1210/endo.139.2.5751

[CR3] Ahmad I, Shukla S, Kumar A et al (2013) Biochemical and molecular mechanisms of N-acetyl cysteine and silymarin-mediated protection against maneb- and paraquat-induced hepatotoxicity in rats. Chem Biol Interact 201(1–3):9–18. 10.1016/j.cbi.2012.10.027 23159886 10.1016/j.cbi.2012.10.027

[CR4] Alabi QK, Akomolafe RO, Olukiran OS et al (2017) The Garcinia kola biflavonoid kolaviron attenuates experimental hepatotoxicity induced by diclofenac. Pathophysiology 24(4):281–290. 10.1016/j.pathophys.2017.07.003 28822616 10.1016/j.pathophys.2017.07.003

[CR5] Allameh A, Niayesh-Mehr R, Aliarab A, Sebastiani G, Pantopoulos K (2023) Oxidative stress in liver pathophysiology and disease. Antioxidants (Basel). 10.3390/antiox12091653 37759956 10.3390/antiox12091653 PMC10525124

[CR6] Altman R, Bosch B, Brune K, Patrignani P, Young C (2015) Advances in NSAID development: evolution of diclofenac products using pharmaceutical technology. Drugs 75(8):859–877. 10.1007/s40265-015-0392-z 25963327 10.1007/s40265-015-0392-z PMC4445819

[CR7] Al-Waeli H, Reboucas AP, Mansour A, Morris M, Tamimi F, Nicolau B (2021) Non-steroidal anti-inflammatory drugs and bone healing in animal models-a systematic review and meta-analysis. Syst Rev 10(1):201. 10.1186/s13643-021-01690-w 34238360 10.1186/s13643-021-01690-w PMC8268344

[CR8] Angulo P, Grandison GA, Fong DG et al (2011) Bone disease in patients with primary sclerosing cholangitis. Gastroenterology 140(1):180–188. 10.1053/j.gastro.2010.10.014 20955707 10.1053/j.gastro.2010.10.014 PMC3043598

[CR9] Aninat C, Piton A, Glaise D et al (2006) Expression of cytochromes P450, conjugating enzymes and nuclear receptors in human hepatoma HepaRG cells. Drug Metab Dispos 34(1):75–83. 10.1124/dmd.105.006759 16204462 10.1124/dmd.105.006759

[CR10] Aspera-Werz RH, Ehnert S, Heid D et al (2018) Nicotine and cotinine inhibit catalase and glutathione reductase activity contributing to the impaired osteogenesis of SCP-1 cells exposed to cigarette smoke. Oxid Med Cell Longev 2018:3172480. 10.1155/2018/3172480 30533170 10.1155/2018/3172480 PMC6250005

[CR11] Bakker AD, Kulkarni RN, Klein-Nulend J, Lems WF (2014) IL-6 alters osteocyte signaling toward osteoblasts but not osteoclasts. J Dent Res 93(4):394–399. 10.1177/0022034514522485 24492932 10.1177/0022034514522485

[CR12] Bazzell KL, Price G, Tu S, Griffin M (1976) Cortisol modification of HeLa 65 alkaline phosphatase. Decreased phosphate content of the induced enzyme. Eur J Biochem. 61(2):493–9. 10.1111/j.1432-1033.1976.tb10044.x 1248469 10.1111/j.1432-1033.1976.tb10044.x

[CR13] Bocker W, Yin Z, Drosse I et al (2008) Introducing a single-cell-derived human mesenchymal stem cell line expressing hTERT after lentiviral gene transfer. J Cell Mol Med 12(4):1347–1359. 10.1111/j.1582-4934.2008.00299.x 18318690 10.1111/j.1582-4934.2008.00299.x PMC3865677

[CR14] Bouju H, Nastold P, Beck B, Hollender J, Corvini PF, Wintgens T (2016) Elucidation of biotransformation of diclofenac and 4′hydroxydiclofenac during biological wastewater treatment. J Hazard Mater 301:443–452. 10.1016/j.jhazmat.2015.08.054 26410273 10.1016/j.jhazmat.2015.08.054

[CR15] Boyce BF, Xing LP (2008) Functions of RANKL/RANK/OPG in bone modeling and remodeling. Arch Biochem Biophys 473(2):139–146. 10.1016/j.abb.2008.03.018 18395508 10.1016/j.abb.2008.03.018 PMC2413418

[CR16] Cottrell J, O’Connor JP (2010) Effect of non-steroidal anti-inflammatory drugs on bone healing. Pharmaceuticals (Basel) 3(5):1668–1693. 10.3390/ph3051668 27713323 10.3390/ph3051668 PMC4034003

[CR17] Cuklev F, Kristiansson E, Fick J, Asker N, Forlin L, Larsson DG (2011) Diclofenac in fish: blood plasma levels similar to human therapeutic levels affect global hepatic gene expression. Environ Toxicol Chem 30(9):2126–2134. 10.1002/etc.599 21688307 10.1002/etc.599

[CR18] Dean L, Kane M (2012) Celecoxib Therapy and CYP2C9 Genotype. In: Pratt VM, Scott SA, Pirmohamed M, Esquivel B, Kattman BL, Malheiro AJ (eds) Medical Genetics Summaries. Bethesda (MD). https://www.ncbi.nlm.nih.gov/books/NBK379478/28520369

[CR19] Degen PH, Dieterle W, Schneider W, Theobald W, Sinterhauf U (1988) Pharmacokinetics of diclofenac and five metabolites after single doses in healthy volunteers and after repeated doses in patients. Xenobiotica 18(12):1449–1455. 10.3109/00498258809042267 3245235 10.3109/00498258809042267

[CR20] Dhage PA, Sharbidre AA (2022) Bimodal behavior of ascorbic acid in musca domestica larvae. Bioint Res Appl Chem 12(4):5199–5216. 10.33263/BRIAC124.51995216

[CR21] Dodwell ER, Latorre JG, Parisini E et al (2010) NSAID exposure and risk of nonunion: a meta-analysis of case-control and cohort studies. Calcif Tissue Int 87(3):193–202. 10.1007/s00223-010-9379-7 20552333 10.1007/s00223-010-9379-7

[CR22] Doke SK, Dhawale SC (2015) Alternatives to animal testing: a review. Saudi Pharm J 23(3):223–229. 10.1016/j.jsps.2013.11.002 26106269 10.1016/j.jsps.2013.11.002 PMC4475840

[CR23] Donato MT, Tolosa L, Gomez-Lechon MJ (2015) Culture and functional characterization of human hepatoma HepG2 cells. Methods Mol Biol 1250:77–93. 10.1007/978-1-4939-2074-7_5 26272135 10.1007/978-1-4939-2074-7_5

[CR24] Ehnert S, van Griensven M, Unger M et al (2018) Co-culture with human osteoblasts and exposure to extremely low frequency pulsed electromagnetic fields improve osteogenic differentiation of human adipose-derived mesenchymal stem cells. Int J Mol Sci 19(4):994. 10.3390/ijms19040994 29584629 10.3390/ijms19040994 PMC5979428

[CR25] Ehnert S, Aspera-Werz RH, Ruoss M et al (2019) Hepatic osteodystrophy-molecular mechanisms proposed to favor its development. Int J Mol Sci 20(10):2555. 10.3390/ijms20102555 31137669 10.3390/ijms20102555 PMC6566554

[CR26] Ehnert S, Rinderknecht H, Aspera-Werz RH, Haussling V, Nussler AK (2020) Use of in vitro bone models to screen for altered bone metabolism, osteopathies, and fracture healing: challenges of complex models. Arch Toxicol 94(12):3937–3958. 10.1007/s00204-020-02906-z 32910238 10.1007/s00204-020-02906-z PMC7655582

[CR27] Fabregat I, Moreno-Caceres J, Sanchez A et al (2016) TGF-beta signalling and liver disease. FEBS J 283(12):2219–2232. 10.1111/febs.13665 26807763 10.1111/febs.13665

[CR28] Feng W, Liu HR, Luo TT et al (2017) Combination of IL-6 and sIL-6R differentially regulate varying levels of RANKL-induced osteoclastogenesis through NF-κB, ERK and JNK signaling pathways. Sci Rep-Uk. 10.1038/srep41411 10.1038/srep41411 PMC5269740 28128332

[CR29] Gaston MS, Simpson AH (2007) Inhibition of fracture healing. J Bone Joint Surg Br 89(12):1553–1560. 10.1302/0301-620X.89B12.19671 18057352 10.1302/0301-620X.89B12.19671

[CR30] Ghezelayagh Z, Zabihi M, Zarkesh I et al (2022) Improved differentiation of hESC-derived pancreatic progenitors by using human fetal pancreatic mesenchymal cells in a micro-scalable three-dimensional co-culture system. Stem Cell Rev Rep 18(1):360–377. 10.1007/s12015-021-10266-z 34586606 10.1007/s12015-021-10266-z

[CR31] Griffin MJ, Ber R (1969) Cell cycle events in the hydrocortisone regulation of alkaline phosphatase in HeLa S3 cells. J Cell Biol 40(2):297–304. 10.1083/jcb.40.2.297 5761919 10.1083/jcb.40.2.297 PMC2107623

[CR32] Gunaydin C, Bilge SS (2018) Effects of nonsteroidal anti-inflammatory drugs at the molecular level. Eurasian J Med 50(2):116–121. 10.5152/eurasianjmed.2018.0010 30002579 10.5152/eurasianjmed.2018.0010 PMC6039135

[CR33] Guo H, Weng W, Zhang S et al (2022) Maqui berry and ginseng extracts reduce cigarette smoke-induced cell injury in a 3D bone co-culture model. Antioxidants (Basel) 11(12):2460. 10.3390/antiox11122460 36552669 10.3390/antiox11122460 PMC9774157

[CR34] Hammour MM, Othman A, Aspera-Werz R et al (2022) Optimisation of the HepaRG cell line model for drug toxicity studies using two different cultivation conditions: advantages and limitations. Arch Toxicol 96(9):2511–2521. 10.1007/s00204-022-03329-8 35748891 10.1007/s00204-022-03329-8

[CR35] Harmer D, Falank C, Reagan MR (2019) Interleukin-6 interweaves the bone marrow microenvironment, bone loss, and multiple myeloma. Front Endocrinol. 10.3389/fendo.2018.00788 10.3389/fendo.2018.00788 PMC6333051 30671025

[CR36] Hatipoglu MG, Inal S, Kabay S et al (2015) The influence of different nonsteroidal anti-inflammatory drugs on alveolar bone in rats: an experimental study. Acta Stomatol Croat 49(4):325–30. 10.15644/asc49/4/8 27688417 10.15644/asc49/4/8 PMC4945340

[CR37] Haussling V, Deninger S, Vidoni L et al (2019) Impact of four protein additives in cryogels on osteogenic differentiation of adipose-derived mesenchymal stem cells. Bioengineering (Basel) 6(3):67. 10.3390/bioengineering6030067 31394780 10.3390/bioengineering6030067 PMC6784125

[CR38] Haussling V, Aspera-Werz RH, Rinderknecht H et al (2021) 3D environment is required in vitro to demonstrate altered bone metabolism characteristic for type 2 diabetics. Int J Mol Sci 22(6):2925. 10.3390/ijms22062925 33805833 10.3390/ijms22062925 PMC8002142

[CR39] Hernandez RK, Do TP, Critchlow CW, Dent RE, Jick SS (2012) Patient-related risk factors for fracture-healing complications in the United Kingdom General Practice Research Database. Acta Orthop 83(6):653–660. 10.3109/17453674.2012.747054 23140093 10.3109/17453674.2012.747054 PMC3555441

[CR40] Hoffmann SA, Muller-Vieira U, Biemel K et al (2012) Analysis of drug metabolism activities in a miniaturized liver cell bioreactor for use in pharmacological studies. Biotechnol Bioeng 109(12):3172–3181. 10.1002/bit.24573 22688505 10.1002/bit.24573

[CR41] Jin X, Li Y, Li J et al (2022) Acute bone damage through liver-bone axis induced by thioacetamide in rats. BMC Pharmacol Toxicol 23(1):29. 10.1186/s40360-022-00568-4 35526079 10.1186/s40360-022-00568-4 PMC9080193

[CR42] Karanikola T, Cheva A, Sarafidou K et al (2022) Effect of diclofenac and simvastatin on bone defect healing-an in vivo animal study. Biomimetics (Basel) 7(4):143. 10.3390/biomimetics7040143 36278700 10.3390/biomimetics7040143 PMC9589953

[CR43] Knospel F, Jacobs F, Freyer N et al (2016) In Vitro model for hepatotoxicity studies based on primary human hepatocyte cultivation in a perfused 3D bioreactor system. Int J Mol Sci 17(4):584. 10.3390/ijms17040584 27092500 10.3390/ijms17040584 PMC4849040

[CR44] Krischak GD, Augat P, Blakytny R, Claes L, Kinzl L, Beck A (2007) The non-steroidal anti-inflammatory drug diclofenac reduces appearance of osteoblasts in bone defect healing in rats. Arch Orthop Trauma Surg 127(6):453–458. 10.1007/s00402-007-0288-9 17245601 10.1007/s00402-007-0288-9

[CR45] Ku EC, Lee W, Kothari HV, Scholer DW (1986) Effect of diclofenac sodium on the arachidonic acid cascade. Am J Med 80(4B):18–23. 10.1016/0002-9343(86)90074-4 3085488 10.1016/0002-9343(86)90074-4

[CR46] Lacey DL, Timms E, Tan HL et al (1998) Osteoprotegerin ligand is a cytokine that regulates osteoclast differentiation and activation. Cell 93(2):165–176. 10.1016/S0092-8674(00)81569-X 9568710 10.1016/s0092-8674(00)81569-x

[CR47] Lee J, Kim J, Lee R et al (2022) Therapeutic strategies for liver diseases based on redox control systems. Biomed Pharmacother. 10.1016/j.biopha.2022.113764 36228367 10.1016/j.biopha.2022.113764

[CR48] Lemieux JM, Wu G, Morgan JA, Kacena MA (2011) DMSO regulates osteoclast development in vitro. In Vitro Cell Dev Biol Anim 47(3):260–267. 10.1007/s11626-011-9385-8 21359822 10.1007/s11626-011-9385-8 PMC3104242

[CR49] Lin J, Schyschka L, Muhl-Benninghaus R et al (2012) Comparative analysis of phase I and II enzyme activities in 5 hepatic cell lines identifies Huh-7 and HCC-T cells with the highest potential to study drug metabolism. Arch Toxicol 86(1):87–95. 10.1007/s00204-011-0733-y 21735230 10.1007/s00204-011-0733-y

[CR50] Liu Z, Han T, Werner H, Rosen CJ, Schaffler MB, Yakar S (2018) Reduced Serum IGF-1 associated with hepatic osteodystrophy is a main determinant of low cortical but not trabecular bone mass. J Bone Miner Res 33(1):123–136. 10.1002/jbmr.3290 28902430 10.1002/jbmr.3290 PMC5771972

[CR51] Lu K, Shi TS, Shen SY et al (2022) Defects in a liver-bone axis contribute to hepatic osteodystrophy disease progression. Cell Metab. 34(3):441-457.e7. 10.1016/j.cmet.2022.02.006 35235775 10.1016/j.cmet.2022.02.006

[CR52] Mazziotti G, Canalis E, Giustina A (2010) Drug-induced osteoporosis: mechanisms and clinical implications. Am J Med 123(10):877–884. 10.1016/j.amjmed.2010.02.028 20920685 10.1016/j.amjmed.2010.02.028

[CR53] McGonigle P, Ruggeri B (2014) Animal models of human disease: challenges in enabling translation. Biochem Pharmacol 87(1):162–171. 10.1016/j.bcp.2013.08.006 23954708 10.1016/j.bcp.2013.08.006

[CR54] McMillian MK, Li L, Parker JB et al (2002) An improved resazurin-based cytotoxicity assay for hepatic cells. Cell Biol Toxicol 18(3):157–173. 10.1023/a:1015559603643 12083422 10.1023/a:1015559603643

[CR55] Mechcatie E (2010) FDA highlights liver risks with diclofenac. Caring for the Ages 11(4):23. 10.1016/S1526-4114(10)60108-4

[CR56] Mekala NK, Baadhe RR, Rao Parcha S, Prameela Devi Y (2013) Enhanced proliferation and osteogenic differentiation of human umbilical cord blood stem cells by L-ascorbic acid, in vitro. Curr Stem Cell Res Ther 8(2):156–162. 10.2174/1574888x11308020006 23140501 10.2174/1574888x11308020006

[CR57] Menger MM, Stief M, Scheuer C et al (2023) Diclofenac, a NSAID, delays fracture healing in aged mice. Exp Gerontol 178:112201. 10.1016/j.exger.2023.112201 37169100 10.1016/j.exger.2023.112201

[CR58] Mihara M, Hashizume M, Yoshida H, Suzuki M, Shiina M (2012) IL-6/IL-6 receptor system and its role in physiological and pathological conditions. Clin Sci 122(3–4):143–159. 10.1042/Cs20110340 10.1042/CS20110340 22029668

[CR59] Miyatake S, Ichiyama H, Kondo E, Yasuda K (2009) Randomized clinical comparisons of diclofenac concentration in the soft tissues and blood plasma between topical and oral applications. Br J Clin Pharmacol 67(1):125–129. 10.1111/j.1365-2125.2008.03333.x 19133062 10.1111/j.1365-2125.2008.03333.x PMC2668093

[CR60] Rose S, Cuvellier M, Ezan F et al (2022) DMSO-free highly differentiated HepaRG spheroids for chronic toxicity, liver functions and genotoxicity studies. Arch Toxicol 96(1):243–258. 10.1007/s00204-021-03178-x 34762139 10.1007/s00204-021-03178-x

[CR61] Ruoss M, Damm G, Vosough M et al (2019) Epigenetic modifications of the liver tumor cell line HepG2 increase their drug metabolic capacity. Int J Mol Sci 20(2):347. 10.3390/ijms20020347 30654452 10.3390/ijms20020347 PMC6358789

[CR62] Scallion R, Moore KA (2009) Effects of food intake on the pharmacokinetics of diclofenac potassium soft gelatin capsules: a single-dose, randomized, two-way crossover study. Clin Ther 31(10):2233–2241. 10.1016/j.clinthera.2009.10.001 19922894 10.1016/j.clinthera.2009.10.001

[CR63] Schyschka L, Sanchez JJ, Wang Z et al (2013) Hepatic 3D cultures but not 2D cultures preserve specific transporter activity for acetaminophen-induced hepatotoxicity. Arch Toxicol 87(8):1581–1593. 10.1007/s00204-013-1080-y 23728527 10.1007/s00204-013-1080-y

[CR64] Sens C, Altrock E, Rau K et al (2017) An O-Glycosylation of fibronectin mediates hepatic osteodystrophy through alpha4beta1 Integrin. J Bone Miner Res 32(1):70–81. 10.1002/jbmr.2916 27427791 10.1002/jbmr.2916

[CR65] Singh P, Rastogi S, Bansal M et al (2015) A prospective study to assess the levels of interleukin-6 following administration of diclofenac, ketorolac and tramadol after surgical removal of lower third molars. J Maxillofac Oral Surg 14(2):219–225. 10.1007/s12663-013-0609-1 26028838 10.1007/s12663-013-0609-1 PMC4444661

[CR66] Sousa B, Lopes J, Leal A et al (2021) Specific glutathione-S-transferases ensure an efficient detoxification of diclofenac in *Solanum lycopersicum* L. plants. Plant Physiol Biochem 168:263–271. 10.1016/j.plaphy.2021.10.019 34666279 10.1016/j.plaphy.2021.10.019

[CR67] Spirlandeli AL, Dick-de-Paula I, Zamarioli A et al (2017) Hepatic osteodystrophy: the mechanism of bone loss in hepatocellular disease and the effects of pamidronate treatment. Clinics (Sao Paulo) 72(4):231–237. 10.6061/clinics/2017(04)07 28492723 10.6061/clinics/2017(04)07 PMC5401620

[CR68] Sriuttha P, Sirichanchuen B, Permsuwan U (2018) Hepatotoxicity of nonsteroidal anti-inflammatory drugs: a systematic review of randomized controlled trials. Int J Hepatol 2018:5253623. 10.1155/2018/5253623 29568654 10.1155/2018/5253623 PMC5820561

[CR69] Tannirandorn P, Epstein S (2000) Drug-induced bone loss. Osteoporos Int 11(8):637–659. 10.1007/s001980070062 11095167 10.1007/s001980070062

[CR70] Tateishi Y, Ohe T, Ogawa M, Takahashi K, Nakamura S, Mashino T (2020) Development of novel diclofenac analogs designed to avoid metabolic activation and hepatocyte toxicity. ACS Omega 5(50):32608–32616. 10.1021/acsomega.0c04942 33376898 10.1021/acsomega.0c04942 PMC7758955

[CR71] Thai PN, Ren L, Xu W et al (2023) Chronic diclofenac exposure increases mitochondrial oxidative stress, inflammatory mediators, and cardiac dysfunction. Cardiovasc Drugs Ther 37(1):25–37. 10.1007/s10557-021-07253-4 34499283 10.1007/s10557-021-07253-4 PMC8904649

[CR72] Todd PA, Sorkin EM (1988) Diclofenac sodium. A reappraisal of its pharmacodynamic and pharmacokinetic properties, and therapeutic efficacy. Drugs 35(3):244–85. 10.2165/00003495-198835030-00004 3286213 10.2165/00003495-198835030-00004

[CR73] Udagawa N, Koide M, Nakamura M et al (2021) Osteoclast differentiation by RANKL and OPG signaling pathways. J Bone Miner Metab 39(1):19–26. 10.1007/s00774-020-01162-6 33079279 10.1007/s00774-020-01162-6

[CR74] Vairetti M, Di Pasqua LG, Cagna M, Richelmi P, Ferrigno A, Berardo C (2021) Changes in glutathione content in liver diseases: an update. Antioxidants (Basel). 10.3390/antiox10030364 33670839 10.3390/antiox10030364 PMC7997318

[CR75] Weng W, Haussling V, Aspera-Werz RH et al (2020) Material-dependent formation and degradation of bone matrix-comparison of two cryogels. Bioengineering (Basel) 7(2):52. 10.3390/bioengineering7020052 32517006 10.3390/bioengineering7020052 PMC7378764

[CR76] Wu Q, Zhou XK, Huang DQ, Ji YC, Kang FW (2017) IL-6 enhances osteocyte-mediated osteoclastogenesis by promoting JAK2 and RANKL activity. Cell Physiol Biochem 41(4):1360–1369. 10.1159/000465455 28278513 10.1159/000465455

[CR77] Yang C, Madhu V, Thomas C et al (2015) Inhibition of differentiation and function of osteoclasts by dimethyl sulfoxide (DMSO). Cell Tissue Res 362(3):577–585. 10.1007/s00441-015-2245-1 26224539 10.1007/s00441-015-2245-1

[CR78] Yasar U, Eliasson E, Forslund-Bergengren C et al (2001) The role of CYP2C9 genotype in the metabolism of diclofenac in vivo and in vitro. Eur J Clin Pharmacol 57(10):729–735. 10.1007/s00228-001-0376-7 11829203 10.1007/s00228-001-0376-7

[CR79] Zahmatkesh E, Othman A, Braun B et al (2022) In vitro modeling of liver fibrosis in 3D microtissues using scalable micropatterning system. Arch Toxicol 96(6):1799–1813. 10.1007/s00204-022-03265-7 35366062 10.1007/s00204-022-03265-7

[CR80] Zhao MZ, Ma JS, Li M et al (2021) Cytochrome P450 enzymes and drug metabolism in humans. Int J Mol Sci. 10.3390/ijms222312808 34884615 10.3390/ijms222312808 PMC8657965

[CR81] Zhu S, Haussling V, Aspera-Werz RH et al (2020) Bisphosphonates reduce smoking-induced osteoporotic-like alterations by regulating RANKL/OPG in an osteoblast and osteoclast co-culture model. Int J Mol Sci 22(1):53. 10.3390/ijms22010053 33374546 10.3390/ijms22010053 PMC7793101

