# Supplementary material for: Establishment of a human 3D in vitro liver-bone model as a potential system for drug toxicity screening
Source: Arch Toxicol. 2024 Nov 6;99(1):333–56. doi: 10.1007/s00204-024-03899-9 (PMC11742461; doi:10.1007/s00204-024-03899-9)
Supplement: Supplementary file 1 — Supplementary file1 (DOCX 200 KB) [file 204_2024_3899_MOESM1_ESM.docx]

**Fig. S1**
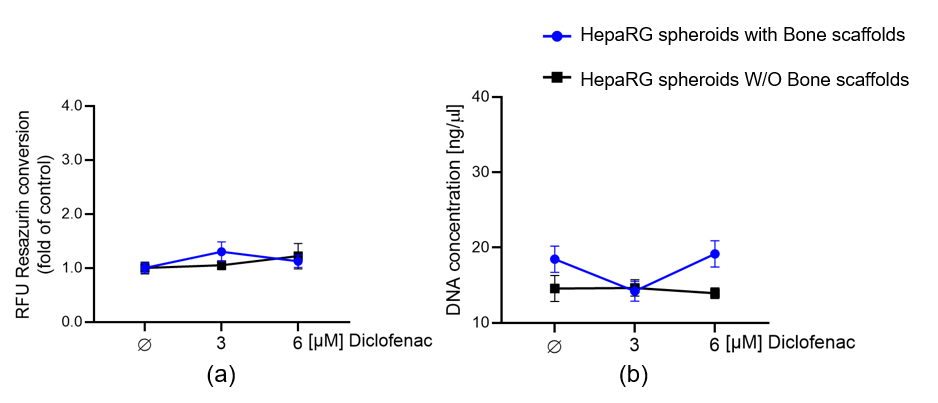


**Sup. Figure 1 HepaRG spheroids and liver-bone system were stimulated with 3 and 6 µM diclofenac for up to 21 days. Diclofenac exposed to HepaRG spheroids with or without bone scaffolds, hepatocyte viability assessment by (a) Resazurin conversion (mitochondrial activity) and (b) DNA content activity on day 21. The Kruskal–Wallis test followed by Dunn’s multiple comparison test was used to determine statistical differences. Data are presented as means ± SEM. N = 3, n = 2.**

**Fig. S2**
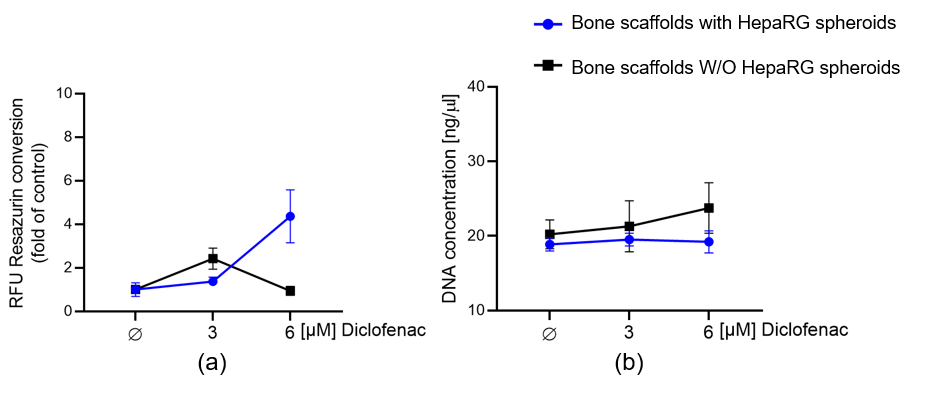


**Sup. Figure 2 Bone scaffolds and liver-bone system were stimulated with 3 and 6 µM diclofenac for up to 21 days. Diclofenac exposed to Bone scaffolds with or without HepaRG spheroids, bone cells viability assessment by (a) Resazurin conversion (mitochondrial activity) and (b) DNA content activity on day 21. The Kruskal–Wallis test followed by Dunn’s multiple comparison test was used to determine statistical differences. Data are presented as means ± SEM. N = 3, n = 3.**

**Fig. S3
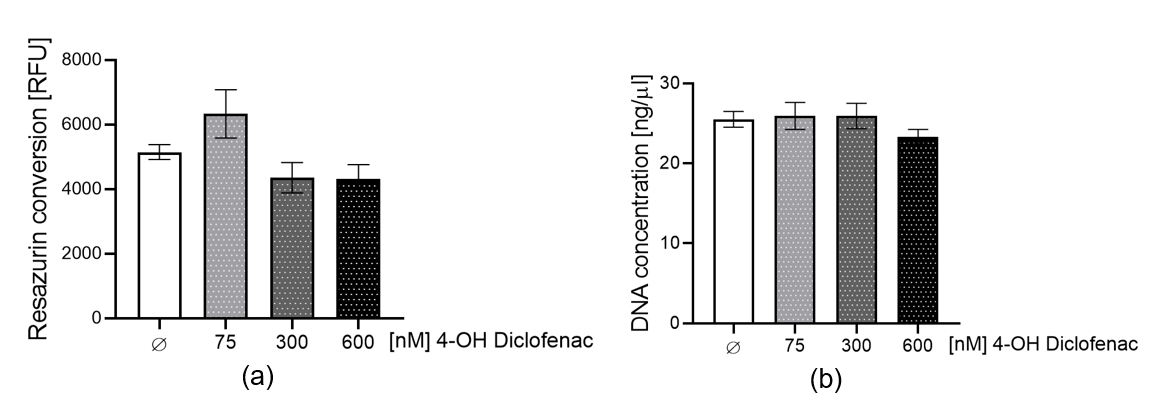
**

**Sup. Figure 3 Bone scaffolds were stimulated with 75, 300, and 600 nM 4-OH diclofenac for up to 21 days. Bone cell viability assessment by (a) Resazurin conversion (mitochondrial activity) and (b) DNA content activity on day 21. The Kruskal–Wallis test followed by Dunn’s multiple comparison test was used to determine statistical differences. Data are presented as means ± SEM. N = 3, n = 3.**
